# Gestational Weight Gain and Its Effects on Maternal and Neonatal Outcome in Women With Twin Pregnancies: A Systematic Review and Meta-Analysis

**DOI:** 10.3389/fped.2021.674414

**Published:** 2021-07-09

**Authors:** Wei Zhong, Xiaojiao Fan, Fang Hu, Meiqin Chen, Fanshu Zeng

**Affiliations:** ^1^Department of Obstetrics, Chengdu Traditional Chinese Medicine University Affiliated Hospital, Chengdu, China; ^2^Department of Pediatrics, Chengdu Traditional Chinese Medicine University Affiliated Hospital, Chengdu, China; ^3^Department of Emergency, Chengdu Traditional Chinese Medicine University Affiliated Hospital, Chengdu, China

**Keywords:** gestational weight gain, twin pregnancy, maternal outcomes, neonatal outcomes, meta-analysis

## Abstract

**Background:** The incidence of twin pregnancies has risen recently. Such pregnancies are associated with an increased risk for poor maternal and infant outcomes. Gestational weight gain, particularly in singleton pregnancies, has been well-linked with maternal and infant outcomes. The aim of the current meta-analysis was to evaluate the effects of gestational weight gain on maternal and fetal outcomes in women with twin pregnancies.

**Methods:** A systematic search was conducted using the PubMed, Scopus, and Google Scholar databases. Studies, either retrospective or prospective in design, evaluating the effects of gestational weight gain (defined using Institute of Medicine (IOM) guidelines) maternal and/or fetal/neonatal outcomes in women with twin pregnancies were included. Statistical analysis was performed using STATA software.

**Results:** Eleven studies were included in the meta-analysis. Mothers with inadequate weight gain had increased risk for gestational diabetes mellitus (OR 1.19; 95% CI: 1.01, 1.40) and decreased risk for gestational hypertension (OR 0.58; 95% CI: 0.49, 0.68) and cesarean section (OR 0.94; 95% CI: 0.93, 0.96). Neonates born to mothers with inadequate weight gain were susceptible to increased risk for preterm delivery (OR 1.17; 95% CI: 1.03, 1.34), very preterm delivery (gestational age <32 weeks) (OR 1.84; 95% CI: 1.36, 2.48), small for gestational age status (OR 1.41; 95% CI: 1.15, 1.72), low birth weight status (<2,500 g) (OR 1.27; 95% CI: 1.17, 1.38), and neonatal intensive care unit (NICU) admission (OR 1.16; 95% CI: 1.08, 1.24). The pooled findings indicate an increased risk for gestational hypertension (OR 1.82; 95% CI: 1.60, 2.06) and cesarean section (OR 1.07; 95% CI: 1.05, 1.08) among mothers with excessive weight gain. Neonates born to mothers with excessive weight gain were susceptible to increased risk for preterm delivery and very preterm delivery, but were associated with a decreased risk for low birth weight status and small for gestational age status.

**Conclusions:** Gestational weight gain in twin pregnancy, either lower or higher than IOM recommended guidelines, is associated with poor maternal and neonatal outcomes. Our findings call for incorporating counseling on optimal weight gain during pregnancy as part of routine antenatal visits.

## Introduction

The incidence of twin pregnancies has increased over the last 2–3 decades (1, 2), and given that twin pregnancies have been linked with increased risk of poor maternal and infant outcomes (3–6), this could potentially emerge as a substantial clinical and public health problem. Furthermore, a wide array of obstetric and perinatal complications are linked to multiple gestation, including hypertensive disorders, gestational diabetes, prematurity, low birth weight, small sizes for gestational age, and perinatal death (3–8). In particular, prematurity is one of the common outcomes of multiple gestation and adds a substantial burden on the health care system (9, 10). According to a reliable estimate, while twin pregnancies constitute only around 3–4% of all birth globally, they contribute to more than one-fifth (20%) of the burden of preterm births (11, 12).

Among a multitude of factors that could potentially impact pregnancy outcomes, one of the readily modifiable factors is gestational weight gain (13, 14). The Institute of Medicine (IOM) has developed recommendations for singleton and twin pregnancies, determining optimum weight gain on the basis of the mother's pre-pregnancy body mass index (BMI) (15, 16). For the twin pregnancies, the IOM guidelines suggest the optimal weight gain to be 16.8–24.5 kg (37–54 lb) or 0.45–0.66 kg/weeks for normal pre-pregnancy BMI (18.5–24.9 kg/m^2^), 14.1–22.7 kg (31–50 lb) or 0.38–0.61 kg/weeks for overweight BMI (25–29.9 kg/m^2^) and 11.3–19.1 kg (25–42 lb) or 0.31–0.52 kg/weeks for obese BMI (≥30 kg/m^2^) mothers (15, 16). A recently published meta-analysis using 23 studies documented an increased risk of small for gestational age (SGA) status and preterm birth in mothers with inadequate gestational weight gain (13). Furthermore, the review noted a 34% lower risk of SGA and 23% lower risk of preterm birth in mothers with excessive gestational weight gain (13), although this was countered by an increased risk of cesarean delivery.

Despite this work and the guidelines that have been established as a result, various gaps in our understanding still require further research. To date, several studies have analyzed the influence of high and low gestational weight gain (relative to IOM guidelines) in women with twin pregnancies. However, no study has attempted to pool and analyze this evidence. Thus, the aim of the current study was to conduct a meta-analysis to evaluate maternal and fetal outcomes in women with twin pregnancies depending on gestational weight gain.

## Materials and Methods

### Search Strategy

This meta-analysis was conducted according to Preferred Reporting Items for Systematic Reviews and Meta-Analyses (PRISMA) guidelines. Three academic databases (PubMed, Scopus, and Google Scholar) were systematically searched for English language publications made available prior to 31st January 2021. The search strategy included medical topic heading (MeSH) terminology and free text words (Supplementary Table 1). Studies involving twin pregnancies that evaluated maternal and/or fetal/neonatal outcomes based on gestational weight gain were marked for potential inclusion. Reference sections for included studies were also screened for additional potential study candidates.

### Selection Criteria and Methods

The literature screen was conducted by two experts. Potential candidate articles were first examined to remove duplicates, following which full texts were reviewed and evaluated. Disputes concerning study inclusion were resolved via discussion between all study authors.

### Inclusion Criteria

Studies must have been either retrospective record-based or prospective in design to be considered for inclusion. Included studies must have examined the relationship between maternal gestational weight gain during pregnancy and maternal and fetal/neonatal outcomes of interest such as gestational diabetes mellitus, gestational hypertension/pre-eclampsia, premature rupture of membranes, cesarean delivery, postpartum hemorrhage, preterm delivery, small for gestational age state, low birth weight, and admission to neonatal intensive care unit (NICU).

#### Exclusion Criteria

Case-reports or review articles were excluded. Also, studies that did not provide data on the above listed outcomes of interest were excluded.

### Data Extraction and Quality Assessment

Data from included studies was extracted and compiled by two separate individuals. The following data was extracted: study identification information, study setting, design characteristics, operational definition of adequate gestational weight gain, sample size, comparison groups, and main findings. The quality of all included studies was assessed using the Newcastle-Ottawa Quality Assessment Scale (17).

### Statistical Analysis

This meta-analysis was conducted using STATA software (version 13.0). Effect sizes were reported as pooled odds ratios with 95% confidence intervals (CIs) for categorical outcomes. For continuous outcomes, effect sizes were reported as weighted mean differences (WMDs). Since the effect of gestational weight gain on birth outcomes was affected by maternal pre-pregnancy BMI, subgroup analysis was done based on maternal pre-pregnancy body mass index. Maternal pre-pregnancy BMI was categorized as normal (18.5–24.9 kg/m^2^); overweight (25–29.9 kg/m^2^), or obese (BMI ≥ 30 kg/m^2^). *I*^2^ was used as a measure to denote heterogeneity. In instances where *I*^2^ exceeded ~40%, a random effects model was used (18). For reporting statistical significance, a *p* < 0.05 was considered. Egger's test was employed to assess for the presence or absence of publication bias. Bias assessment was further supported by visual inspection of funnel plots.

## Results

### Selection of Articles, Study Characteristics, and Quality of Included Studies

**Figure 1:**
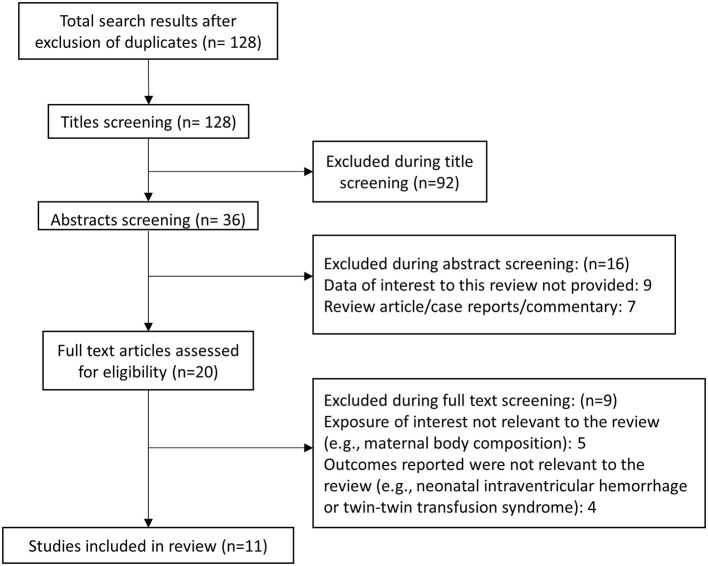
Study inclusion selection process.

Literature search revealed 128 unique studies for consideration (Figure 1). Additional screening resulted in the exclusion of 92 studies. Out of the remaining 36 studies, another 16 were excluded after abstract review, while another nine were removed after detailed review. A final total of 11 articles were included in this meta-analysis (Supplementary Table 2). (19–29). All included studies used Institute of Medicine (IOM) guidelines to classify adequacy of gestational weight gain according to the pre-pregnancy maternal body mass index (BMI). All included studies performed retrospective analysis of previously collected data. Newcastle-Ottawa Quality Assessment Scale analysis indicated that all included studies were of good quality (Supplementary Table 3).

### Overall Pooled Analysis Results

#### Inadequate/Low Weight Gain Compared to Adequate Weight Gain

##### Maternal Outcomes

**Figure 2:**
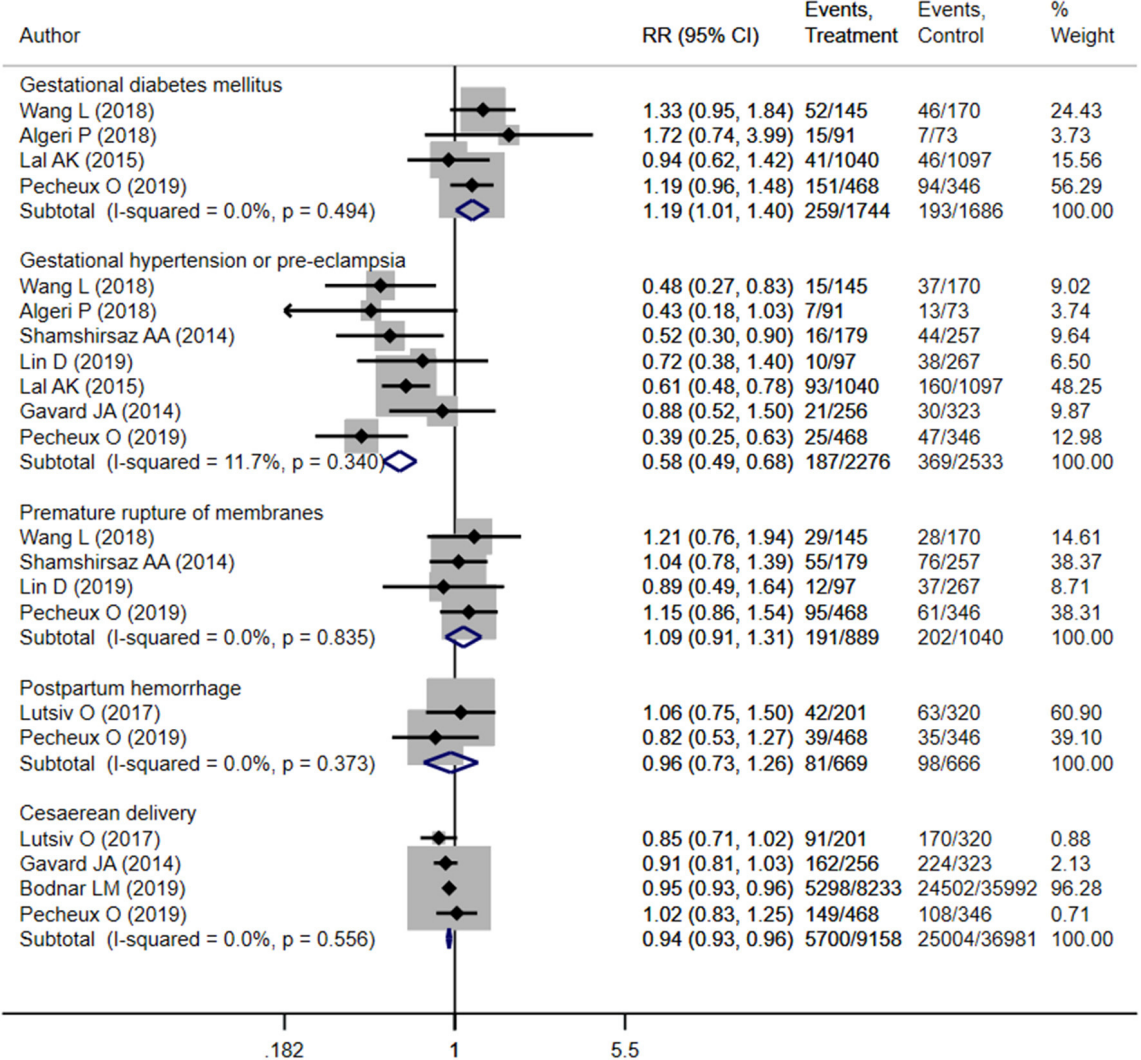
Pooled analysis of inadequate or adequate pregnancy weight gain effects on maternal health outcomes.

Pooled analysis indicated that mothers with inadequate weight gain had increased risk of gestational diabetes mellitus (OR 1.19; 95% CI: 1.01, 1.40; *I*^2^ = 0.0%; *N* = 4), compared to those with adequate weight gain (Figure 2). However, those with inadequate weight gain had lower risk of gestational hypertension (OR 0.58; 95% CI: 0.49, 0.68; *I*^2^ = 11.7%; *N* = 7) and cesarean section (OR 0.94; 95% CI: 0.93, 0.96; *I*^2^ = 0.0%; *N* = 4). Risk of premature membrane rupture (OR 1.09; 95% CI: 0.91, 1.31; *I*^2^ = 0.0%; *N* = 4) or postpartum hemorrhage (OR 0.96; 95% CI: 0.73, 1.26; *I*^2^ = 0.0%; *N* = 2) did not differ significantly between mothers with adequate and inadequate weight gain (Figure 2). There was no evidence of publication bias using Egger's test and a funnel plot (Supplementary Figure 1, *P* = 0.71).

##### Neonatal Outcomes

**Figure 3:**
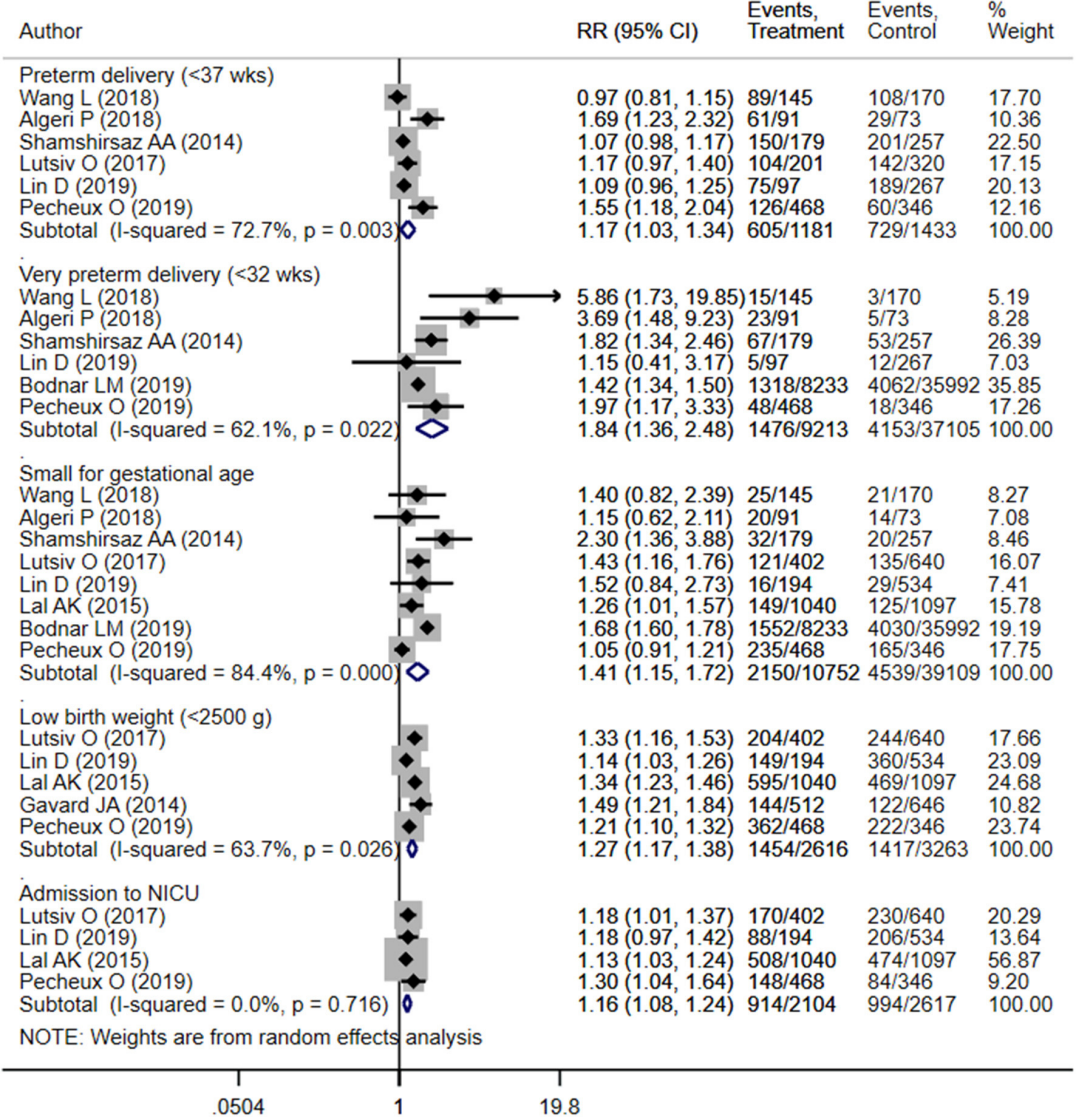
Pooled analysis of inadequate or adequate pregnancy weight gain effects on neonatal health outcomes.

**Figure 4:**
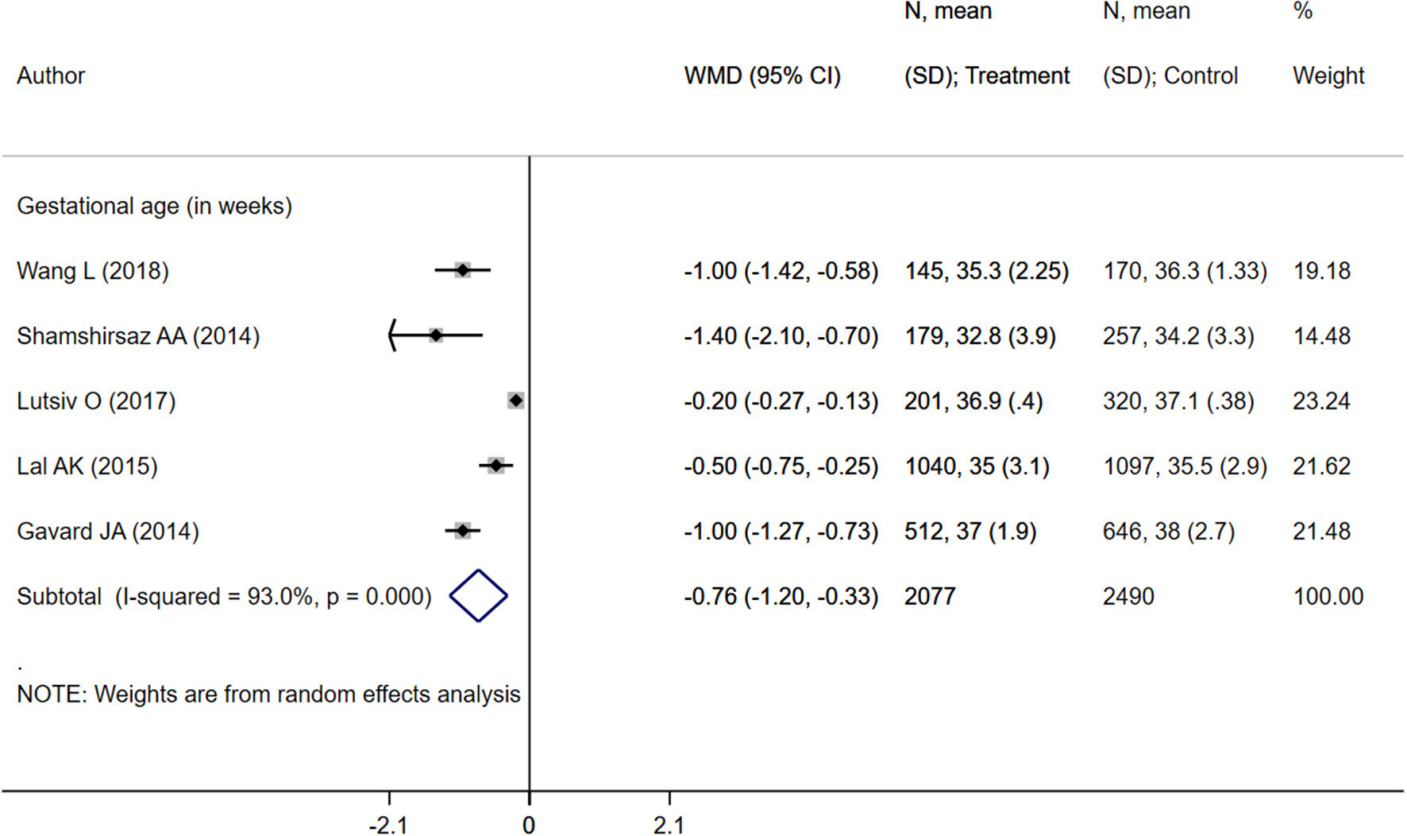
Pooled analysis of inadequate or adequate pregnancy weight gain effects on gestational age.

**Figure 5:**
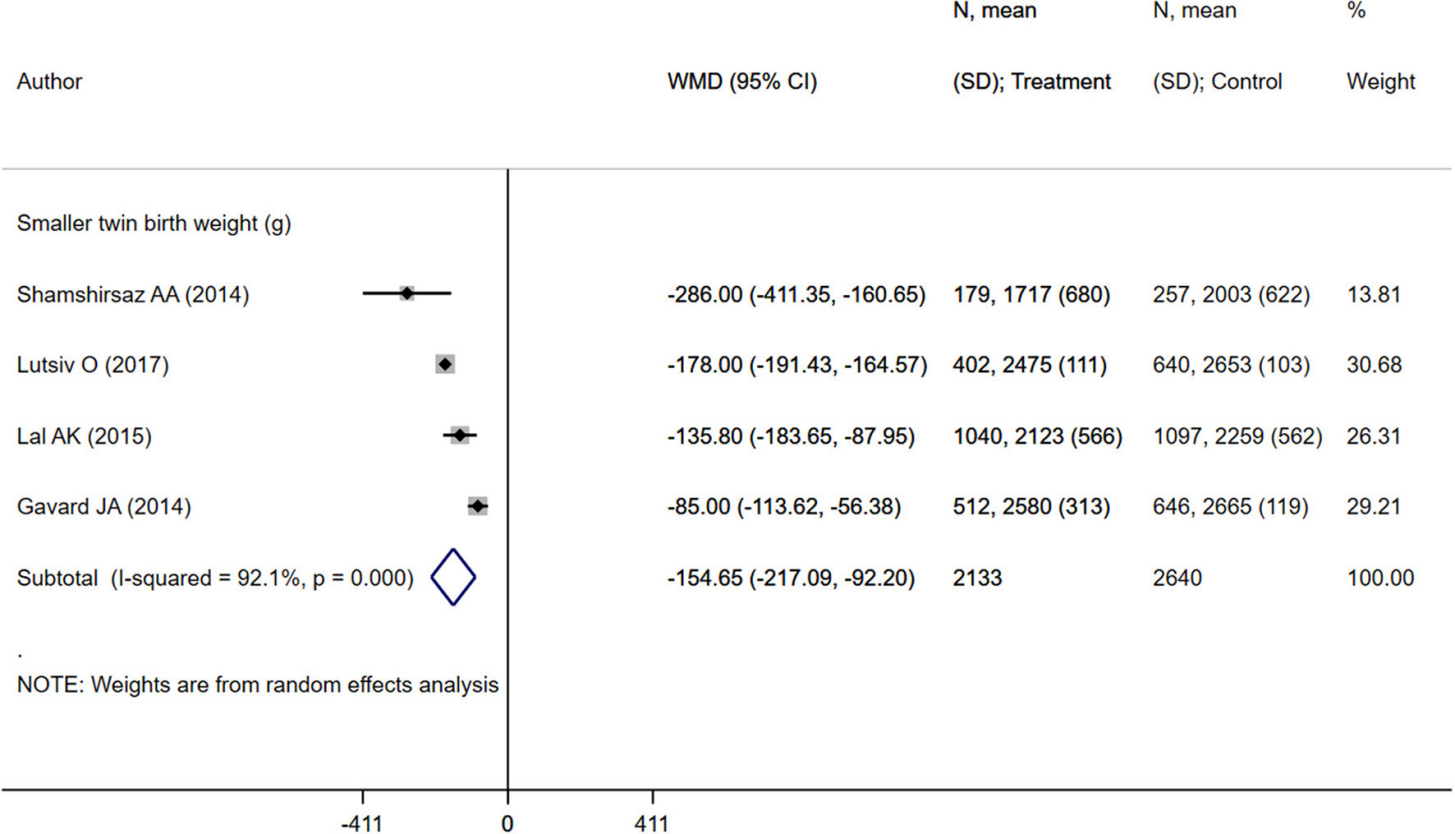
Pooled analysis of inadequate or adequate pregnancy weight gain effects on maternal health outcomes mean birth weight.

Children born to mothers with inadequate weight gain presented an increased risk of preterm delivery (OR 1.17; 95% CI: 1.03, 1.34; *I*^2^ = 72.7%; *N* = 6), very preterm delivery (gestational age <32 weeks) (OR 1.84; 95% CI: 1.36, 2.48; *I*^2^ = 62.1%; *N* = 6), small for gestational age status (OR 1.41; 95% CI: 1.15, 1.72; *I*^2^ = 84.4%; *N* = 8), low birth weight (birth weight <2,500 g) (OR 1.27; 95% CI: 1.17, 1.38; *I*^2^ = 63.7%; *N* = 5), and NICU admission (OR 1.16; 95% CI: 1.08, 1.24; *I*^2^ = 0.0%; *N* = 4) compared to those born to mothers with adequate weight gain (Figure 3). There was no evidence of publication bias using Egger's test and a funnel plot (Supplementary Figure 2, *P* = 0.43). Neonates born to mothers with inadequate weight gain had lower gestational ages (in weeks) (WMD −0.76; 95% CI: −1.20, −0.33; *I*^2^ = 93.0%; *N* = 5) and mean birth weights (in grams) (WMD −154.7; 95% CI: −217.1, −92.2; *I*^2^ = 92.1%; *N* = 4) compared to those born to mothers with adequate weight gain (Figures 4, 5).

#### Excessive Weight Gain Compared to Adequate Weight Gain

##### Maternal Outcomes

**Figure 6:**
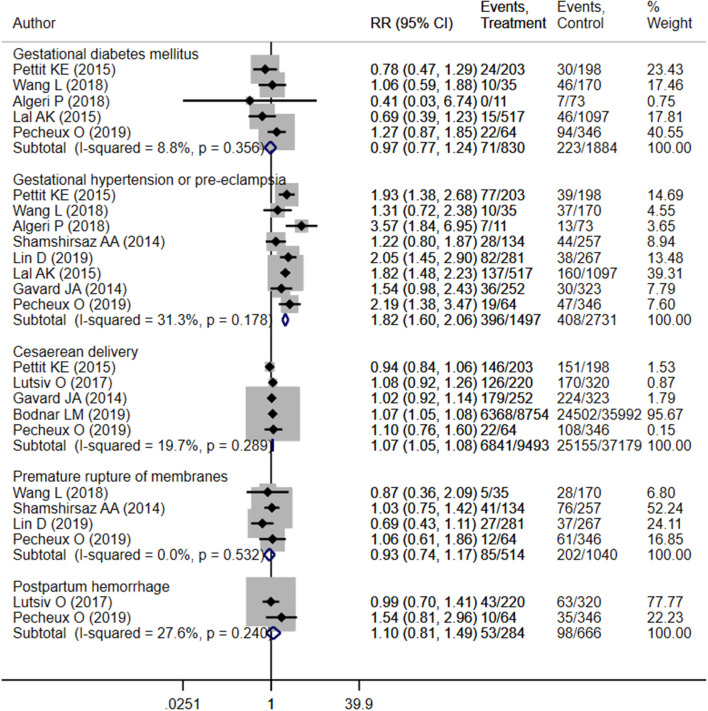
Pooled analysis of excessive or adequate pregnancy weight gain effects on maternal health outcomes.

The findings indicated an increased risk of gestational hypertension (OR 1.82; 95% CI: 1.60, 2.06; *I*^2^ = 31.3%; *N* = 8) and cesarean section (OR 1.07; 95% CI: 1.05, 1.08; *I*^2^ = 19.7%; *N* = 5) among pregnant mothers with excessive weight gain compared to those with adequate gain (Figure 6). Risk of gestational diabetes mellitus (OR 0.97; 95% CI: 0.77, 1.24; *I*^2^ = 8.8%; *N* = 5), premature membrane rupture (OR 0.93; 95% CI: 0.74, 1.17; *I*^2^ = 0.0%; *N* = 4), and postpartum hemorrhage (OR 1.10; 95% CI: 0.81, 1.49; *I*^2^ = 27.6%; *N* = 2) did not differ significantly between mothers with adequate and excessive weight gain (Figure 6). There was no evidence of publication bias (Supplementary Figure 3, *P* = 0.29).

##### Neonatal Outcomes

**Figure 7:**
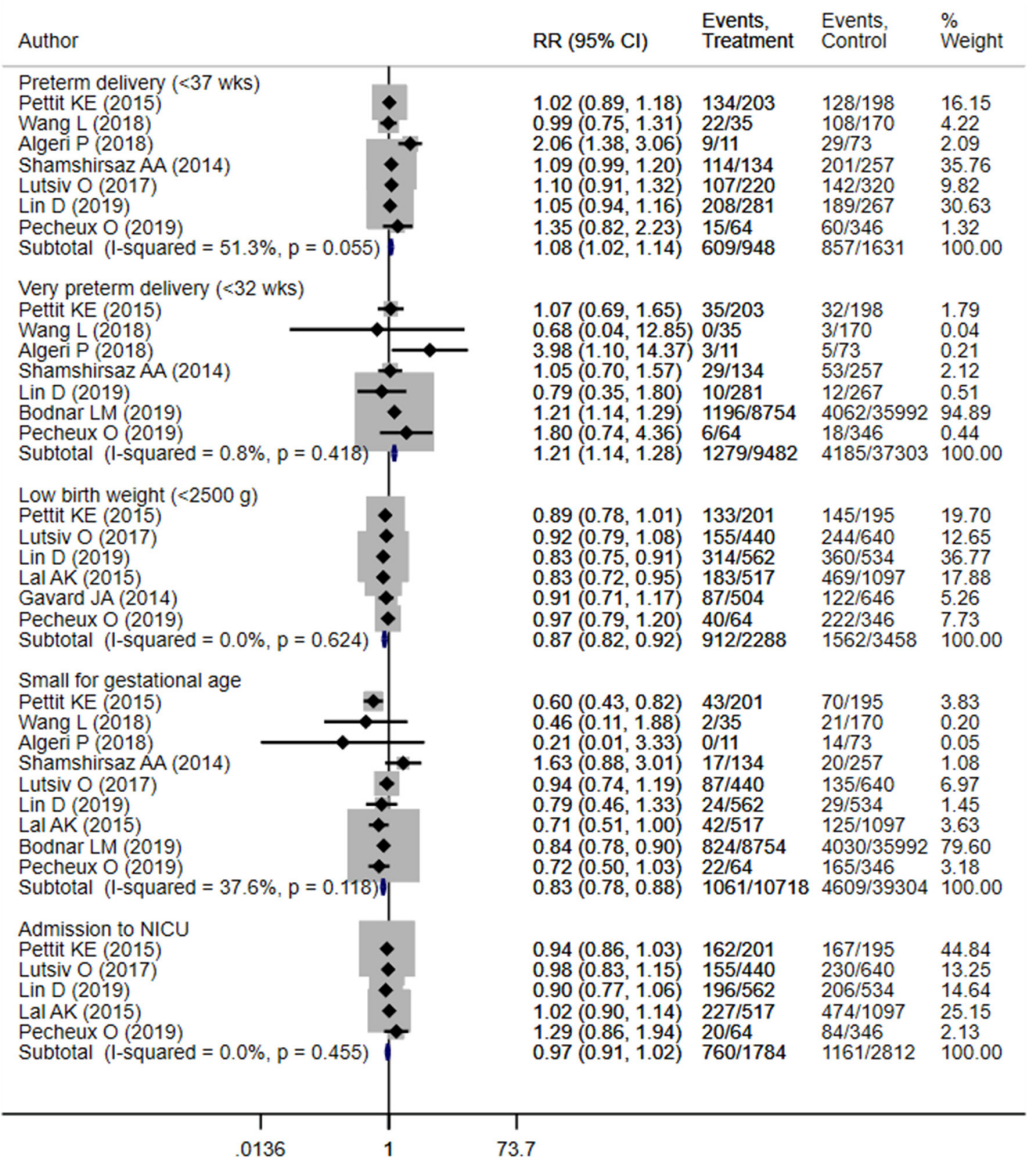
Pooled analysis of excessive or adequate pregnancy weight gain effects on neonatal health outcomes.

**Figure 8:**
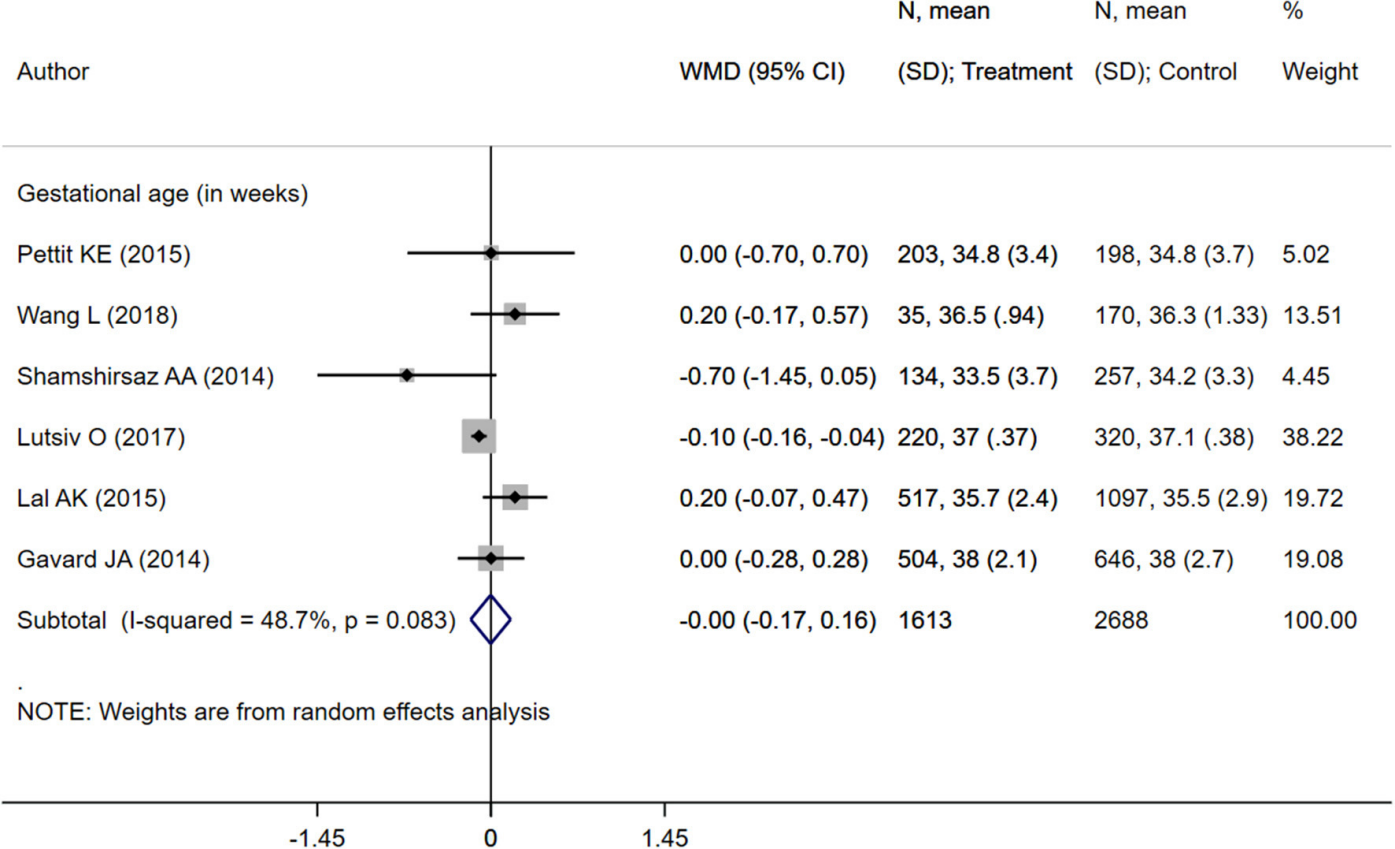
Pooled analysis of excessive or adequate pregnancy weight gain effects on mean gestational age.

**Figure 9:**
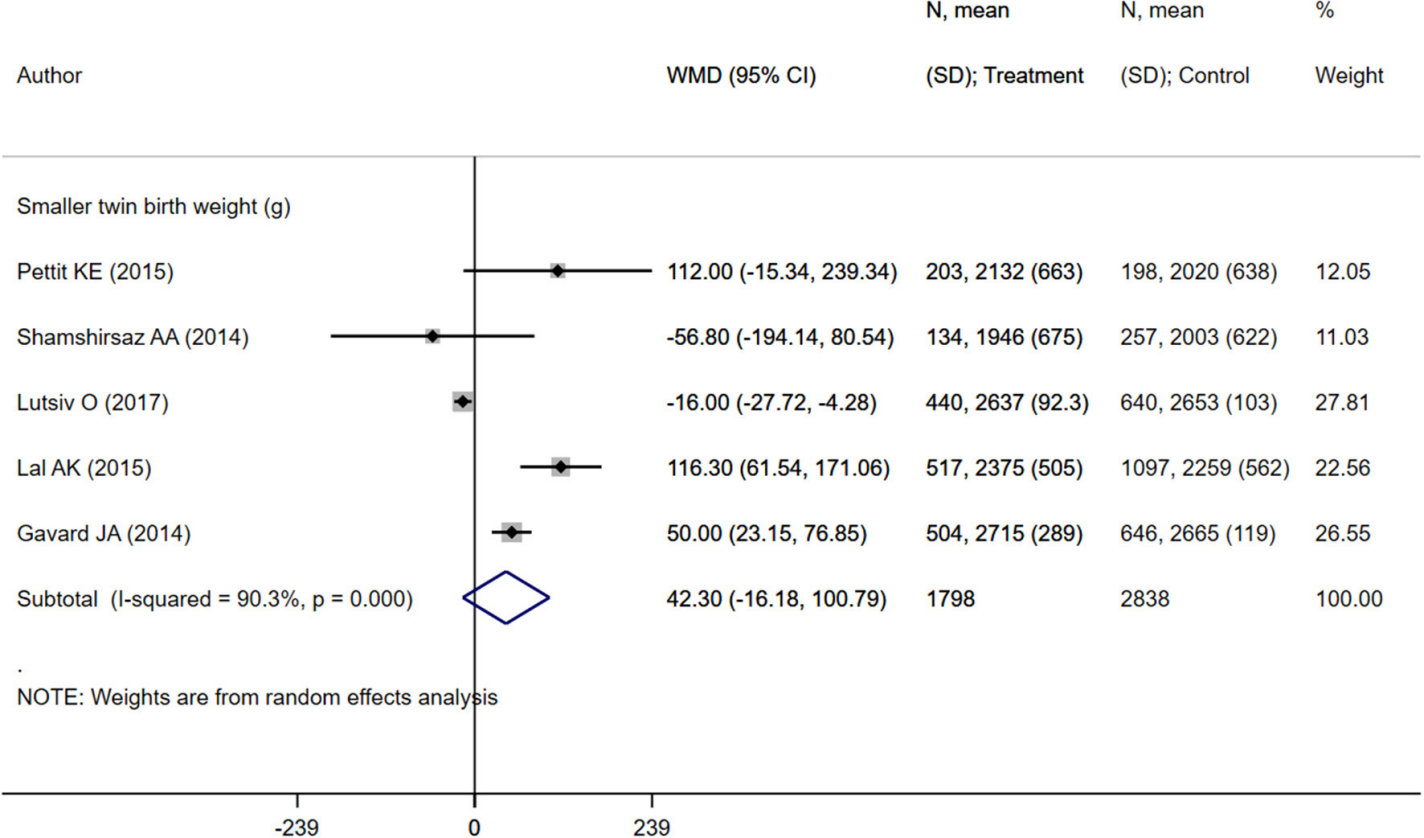
Pooled analysis of excessive or adequate pregnancy weight gain effects on mean birth weight.

Neonates born to mothers with excessive weight gain were at increased risk of preterm delivery (OR 1.08; 95% CI: 1.02, 1.14; *I*^2^ = 51.3%; *N* = 7) and very preterm delivery (gestational age <32 weeks) (OR 1.21; 95% CI: 1.14, 1.28; *I*^2^ = 0.8%; *N* = 7) (Figure 7). However, in such neonates, the risk of low birth weights (OR 0.87; 95% CI: 0.82, 0.92; *I*^2^ = 0.0%; *N* = 6) and small for gestational age status (OR 0.83; 95% CI: 0.78, 0.88; *I*^2^ = 37.6%; *N* = 9) was lower compared to children born to mothers with adequate weight gain (Figure 7). There was no evidence of publication bias (Supplementary Figure 4, *P* = 0.34). Neonates born to mothers with excessive weight gain had similar gestational ages (in weeks) (WMD 0.00; 95% CI: −0.17, 0.16; *I*^2^ = 48.7%; *N* = 6) compared to those born to mothers with adequate weight gain. While the pooled mean birth weight (in grams) (WMD 42.3; 95% CI: −16.2, 100.8; *I*^2^ = 90.3%; *N* = 5) was higher by roughly 42 grams, no statistically significant difference was observed (Figures 8, 9).

#### Subgroup Analysis Based on Maternal Pre-pregnancy BMI

##### Mothers With Normal Pre-pregnancy BMI

Maternal inadequate or low gestational weight gain, compared to adequate weight gain, was associated with an increased risk of small for gestational age status (OR 1.62; 95% CI: 1.30, 2.02; *I*^2^ = 52.6%; *N* = 5), very preterm status (OR 2.38; 95% CI: 1.39, 4.09; *I*^2^ = 76.8%; *N* = 4), low birth weight (OR 1.25; 95% CI: 1.15, 1.37; *I*^2^ = 8.3%; *N* = 2), and NICU admission (OR 1.15; 95% CI: 1.02, 1.30; *N* = 1) (Supplementary Figure 5). However, the risk of gestational hypertension (OR 0.57; 95% CI: 0.44, 0.73; *I*^2^ = 0.0%; *N* = 4) and cesarean delivery (OR 0.93; 95% CI: 0.91, 0.96; *N* = 1) was lower (Supplementary Figure 6).

Maternal excessive gestational weight gain, compared to adequate weight gain, was associated with an increased risk of gestational hypertension (OR 1.92; 95% CI: 1.56, 2.35; *I*^2^ = 44.4%; *N* = 5), cesarean delivery (OR 1.07; 95% CI: 1.04, 1.09; *I*^2^ = 37.6%; *N* = 2), and very preterm delivery (OR 1.20; 95% CI: 1.10, 1.31; *I*^2^ = 0.0%; *N* = 5) (Supplementary Figure 7 and Supplementary Figure 8). However, the risk of small for gestational age status was lower (OR 0.80; 95% CI: 0.72, 0.88; *I*^2^ = 0.0%; *N* = 5). The risk of gestational diabetes mellitus, premature membrane rupture, preterm delivery, low birth weight, and NICU admission did not differ significantly between those with adequate and excessive weight gain.

##### Overweight Mothers (Pre-pregnancy BMI: 25–29.9 KG/M^2^)

Maternal inadequate or low gestational weight gain, compared to adequate weight gain, was associated with an increased risk of small for gestational age status (OR 1.47; 95% CI: 1.04, 2.06; *I*^2^ = 44.8%; *N* = 3) and very preterm status (OR 1.47; 95% CI: 1.31, 1.65; *I*^2^ = 0.0%; *N* = 2) (Supplementary Figure 9). There was a non-statistically significant lower risk of cesarean delivery in mothers with inadequate or low weight gain (OR 0.96; 95% CI: 0.93, 1.00; *N* = 1) (Supplementary Figure 10). The risk of gestational diabetes mellitus, hypertension, premature membrane rupture, preterm delivery, low birth weight, and NICU admission did not differ significantly between those with adequate and inadequate/low gestational weight gain.

Maternal excessive gestational weight gain, compared to adequate weight gain, was associated with increased risk of gestational hypertension (OR 1.65; 95% CI: 1.18, 2.30; *I*^2^ = 0.0%; *N* = 3) and very preterm delivery (OR 1.24; 95% CI: 1.10, 1.39; *I*^2^ = 0.0%; *N* = 3) (Supplementary Figure 11, Supplementary Figure 12). However, the risk of gestational diabetes mellitus reduced (OR 0.21; 95% CI: 0.07, 0.58; *I*^2^ = 0.0%; *N* = 2). The risk of cesarean delivery, premature membrane rupture, preterm delivery, low birth weight, small for gestational age status, and NICU admission was not statistically different between those with adequate and excessive weight gain.

##### Obese Mothers (Pre-pregnancy BMI: ≥30 KG/M^2^)

Maternal inadequate or low gestational weight gain, compared to adequate weight gain, was associated with an increased risk of low birth weight (OR 1.33; 95% CI: 1.06, 1.66; *I*^2^ = 70.9%; *N* = 3), small for gestational age status (OR 1.41; 95% CI: 1.16, 1.71; *I*^2^ = 7.6%; *N* = 3), and very preterm baby (OR 1.32; 95% CI: 1.14, 1.52; *I*^2^ = 2.1%; *N* = 2) (Supplementary Figure 13). There was a reduced risk of cesarean delivery (OR 0.95; 95% CI: 0.93, 0.98; *I*^2^ = 0.0%; *N* = 2) and gestational hypertension (OR 0.62; 95% CI: 0.44, 0.87; *I*^2^ = 30.1%; *N* = 3) in mothers with inadequate or low weight gain (Supplementary Figure 14). The risk of gestational diabetes mellitus, premature membrane rupture, preterm delivery, and NICU admission did not differ significantly between those with adequate and inadequate/low gestational weight gain.

Maternal excessive gestational weight gain, compared to adequate weight gain, was associated with an increased risk of gestational hypertension (OR 1.45; 95% CI: 1.13, 1.86; *I*^2^ = 0.0%; *N* = 4), cesarean delivery (OR 1.07; 95% CI: 1.05, 1.10; *I*^2^ = 0.0%; *N* = 3), and very preterm delivery (OR 1.20; 95% CI: 1.06, 1.34; *I*^2^ = 54.8%; *N* = 3) (Supplementary Figure 15, Supplementary Figure 16). The risk of small for gestational age status was reduced (OR 0.85; 95% CI: 0.74, 0.98; *I*^2^ = 21.5%; *N* = 3). The risk of premature membrane rupture, gestational diabetes, preterm delivery, low birth weight, and NICU admission did not differ significantly between those with adequate and excessive weight gain.

## Discussion

This meta-analysis aimed to evaluate the effects of gestational weight gain on maternal and fetal outcomes in women with twin pregnancies. We herein found an increased risk of negative maternal and fetal outcomes in women experiencing either inadequate or excessive weight gain relative to mothers with optimal gestational weight gain. Mothers with inadequate weight gain were at increased risk for gestational diabetes mellitus, preterm delivery, very preterm delivery, small for gestational age status, low birth weights, and NICU admission. The study also noted an increased risk for gestational hypertension and cesarean section among mothers with excessive weight gain. Neonates born to mothers with excessive weight gain were at increased risk for preterm delivery and very preterm delivery. The findings were largely the same when analysis was conducted based on maternal pre-pregnancy BMI.

A similar meta-analysis, containing 23 studies, was previously conducted for singleton pregnancies (13), finding a higher risk for small for gestational age status and preterm birth when gestational weight gain was below recommended amounts. Moreover, gestational weight gain above baseline was associated with decreased risk for SGA and preterm birth, but increased risk for cesarean delivery (13). Additionally, a large population-based cohort study in United States found that mothers with low gestational weight gain presented increased risk for serious adverse birth effects including maternal and perinatal death (30). The American study also noted an increased risk for poor maternal and fetal outcomes in mothers with excessive gestational weight gain—however, this increased risk was not found in maternal overweight at the time of conception (30).

Low pregnancy weight gain usually indicates the presence of nutritional deficiencies, inadequate expansion of plasma volume and an underlying metabolic state that is supportive of increased risk of infections and inflammation (31, 32). Similarly, it is well-documented that overweight and obesity is a pro-inflammatory state and excessive weight gain during pregnancy is associated with surge in pro-inflammatory cytokines (33–35). All these factors increase the risk of preterm and very preterm delivery. Also, inflammation leads to activation and subsequent expression of several proteins, cytokines and interleukins that suppress the insulin signaling pathways, lead to less sensitivity to insulin and consequent insulin resistance (36). These factors could, in some way, explain the increased risk of preterm delivery and gestational diabetes in women with either low or high gestational weight gain.

Together with the present study, these findings call for prenatal and antenatal programs to support maternal nutrition and encourage optimal gestational weight gain. Pre-conception nutrition programs should be devised that target normal BMIs for expectant mothers. These programs are currently lacking in both developed and developing countries. In addition, mothers should be educated about the health advantages of maintaining normal BMI and achieving optimal weight gain during pregnancy. Lifestyle and nutritional interventions may assist in these endeavors. It is also important to note that the current evidence suggests that glucose intolerance during pregnancy increases the likelihood of developing abnormal glucose tolerance and diabetes later during life (37). In this context, it is important to ensure a healthy lifestyle and nutritional habits starting from early in the pregnancy. Additional to that, myo-inositol, one of the promising molecules that has not only shown to positively affect fertility but also prevention of gestational diabetes mellitus, could be used during pregnancy (38, 39). In a recent Cochrane review, it was found to reduce the incidence of gestational diabetes mellitus by 10–22% (40). Myo-inositol functions by participating in several signaling processes involving insulin and gonadotropins (38–40). Furthermore, women developing abnormal glucose tolerance during pregnancy should closely monitor their glucose levels post-delivery as well.

This study does have several limitations. First, the included studies were mostly conducted in developed settings with minimal representation from low/middle-income demographic groups. This may make the findings difficult to generalize. Second, while included studies reported preterm birth data, no clear distinctions between spontaneous and induced preterm birth were included. Studies also did not mention how gestational age was assessed, which may affect prematurity-related observations. However, the studies reported on small for gestational age based on the standard definition of weight below the 10th percentile for the gestational age (41). The studies did not demarcate elective and emergency cesarean deliveries. Most of the studies included in this review were from developed settings and as part of the standard care in these settings, twin pregnancies are usually delivered through elective cesarean section. Therefore, the observed pooled effect sizes for cesarean section as an outcome may be less reliable. Finally, the findings from this meta-analysis are based on observational data and therefore may not be suitable for establishing causality. Our meta-analysis is limited by the use of studies involving retrospective data procurement and analysis, meaning that data on important confounding factors are not always available. Consequently, the observed associations reported by individual studies may not be accurate, thereby affecting the reliability of the findings of this present meta-analysis.

One important aspect to acknowledge is that the IOM guidelines for adequate weight gain lay a wide range and it is often confusing to decide on what ideal minimum weight gain to aim for. Previously conducted studies, especially in overweight and obese women, have shown that weight gain below the IOM recommendations seemingly does not have any significant negative effect of maternal and neonatal outcomes (14, 42, 43). This may indicate that fetal growth is more important to assess, as opposed to maternal weight gain. Future studies should aim to answer the question of minimum optimal pregnancy weight gain in twin pregnancies across various maternal pre-pregnancy BMI categories.

The findings of the meta-analysis place emphasis on the importance of attaining optimal gestational weight gain in mothers for twin pregnancies. Doing so would possibly aid in preventing adverse maternal and fetal/neonatal outcomes. Counseling on optimal weight gain during pregnancy as part of routine antenatal visits is therefore desirable.

## Data Availability Statement

The original contributions presented in the study are included in the article/Supplementary Materials, further inquiries can be directed to the corresponding author/s.

## Author Contributions

WZ and XF conceived, designed the study, and wrote the paper. FH and MC were involved in literature search, data collection, and analyzed the data. FZ reviewed and edited the manuscript. All authors read and approved the final manuscript.

## Conflict of Interest

The authors declare that the research was conducted in the absence of any commercial or financial relationships that could be construed as a potential conflict of interest.
