# Supplementary material for: Gestational Weight Gain and Its Effects on Maternal and Neonatal Outcome in Women With Twin Pregnancies: A Systematic Review and Meta-Analysis
Source: Front Pediatr. 2021 Jul 9;9:674414. doi: 10.3389/fped.2021.674414 (PMC8298912; doi:10.3389/fped.2021.674414)
Supplement: Supplementary file 1 [file Table_1.doc]

**Table 1.** Key details of studies included in the meta-analysis

| **Author, year of publication** | **Country** | **Study design and study participants** | **Definition of adequate gestational weight gain (GWG)** | **Comparison groups** | **Key outcomes** |
| --- | --- | --- | --- | --- | --- |
| Pettit KE et al (2015) [19] | USA | Retrospective cohort study; women with different pre-pregnancy BMI were included in the analysis   - Normal weight (BMI 18.5–24.9) - Overweight (BMI 25–29.9) - Obese (BMI≥30) | Based on Institute of Medicine (IOM) guidelines   - *Normal BMI:* 0.45-0.66 kg/week - *Overweight*: 0.38-0.61 kg/week - *Obese:* 0.31-0.52 kg/week | Adequate GWG (C) vs. Excessive GWG (I) | **Among all women**  *Gestational diabetes*  I : 24/203 (11.8%); C: 30/198 (15.2%)  *Gestational hypertension, pre-eclampsia or HELLP syndrome*  I: 77/203 (37.9%); C: 39/198 (19.7%)  *Mean (SD) gestation age (in weeks)*  I: 34.8 (3.4); C: 34.8 (3.7)  *Preterm birth (<37 weeks)*  I: 134/203 (66.0%); C: 128/198 (64.6%)  *Very preterm birth (<32 weeks)*  I: 35/203 (17.2%); C: 32/198 (16.2%)  *Caesarean delivery*  I: 146/203 (71.9%); C: 151/198 (76.3%)  *Birth weight <2500 g for at least one twin*  I: 133/201 (66.2%); C: 145/195 (74.4%)  *Birth weight <10th percentile for at least one twin*  I: 43/201 (21.4%); C: 70/195 (35.9%)  *Twin birth weight discordance >20%*  I: 36/201 (17.9%); C: 43/195 (22.1%)  *Smaller twin mean (SD) birth weight (g)*  I: 2132 (663); C: 2020 (638)  *NICU admission for at least one twin*  I: 162/201 (80.6%); C: 167/195 (85.6%)  **Among those with** **pre-pregnancy BMI between 18.5 and 24.9**  *Gestational diabetes*  I : 12/120 (10.0%); C: 16/127 (12.6%)  *Gestational hypertension, pre-eclampsia or HELLP syndrome*  I: 41/120 (34.2%); C: 21/127 (16.5%)  *Mean (SD) gestation age (in weeks)*  I: 34.9 (3.2); C: 35.0 (3.4)  *Preterm birth (<37 weeks)*  I: 80/120 (66.7%); C: 80/127 (63.0%)  *Very preterm birth (<32 weeks)*  I: 20/120 (16.7%); C: 19/127 (15.0%)  *Caesarean delivery*  I: 86/120 (71.7%); C: 94/127 (74.0%)  **Among those with** **pre-pregnancy BMI between 25-29.9**  *Gestational diabetes*  I : 2/46 (4.3%); C: 9/39 (23.1%)  *Gestational hypertension, pre-eclampsia or HELLP syndrome*  I: 23/46 (50.0%); C: 10/39 (25.6%)  *Mean (SD) gestation age (in weeks)*  I: 34.1 (4.0); C: 34.4 (3.9)  *Preterm birth (<37 weeks)*  I: 32/46 (69.6%); C: 27/39 (69.2%)  *Very preterm birth (<32 weeks)*  I: 12/46 (26.1%); C: 7/39 (17.9%)  *Caesarean delivery*  I: 29/46 (63.0%); C: 30/39 (76.9%)  **Among those with** **pre-pregnancy BMI (BMI≥30) i.e., Obese**  *Gestational diabetes*  I : 10/37 (27.0%); C: 5/32 (15.6%)  *Gestational hypertension, pre-eclampsia or HELLP syndrome*  I: 13/37 (35.1%); C: 8/32 (25.0%)  *Mean (SD) gestation age (in weeks)*  I: 35.6 (2.8); C: 34.4 (4.5)  *Preterm birth (<37 weeks)*  I: 22/37 (59.5%); C: 21/32 (65.6%)  *Very preterm birth (<32 weeks)*  I: 3/37 (8.1%); C: 6/32 (18.8%)  *Caesarean delivery*  I: 31/37 (83.8%); C: 27/32 (84.4%) |
| Wang L et al (2018) [20] | China | Retrospective cohort analysis;  Normal, pre-gravid weight Chinese women (prepregnancy BMI 18.5–24.9) | Based on IOM recommendation of total weight gain for women with a prepregnancy BMI of 18.5–24.9 during twin pregnancy to be 16.8–24.5 kg.  Low GWG, adequate GWG and high GWG were defined as a total weight gain less than 16.8 kg, 16.8–24.5 kg, and more than 24.5 kg, respectively. | Adequate GWG (C) vs. low GWG (I 1) and excessive GWG (I2) | *Gestational diabetes mellitus*  I1: 52/145 (35.9%); I2: 10/35 (28.6%); C: 46/170 (27.1%)  *Gestational hypertension or pre-eclampsia*  I1: 15/145 (10.3%); I2: 10/35 (28.6%); C: 37/170 (21.8%)  *Mean (SD) gestational weeks*  I1: 35.29 (2.25) (N=145); I2: 36.50 (0.94) (N=35); C: 36.32 (1.33) (N=170)  *Preterm delivery*  I1: 89/145 (61.4%); I2: 22/35 (62.9%); C: 108/170 (63.5%)  *Very preterm delivery*  I1: 15/145 (10.3%); I2: 0/35 (0.0%); C: 3/170 (1.76%)  *Premature rupture of membrane*  I1: 29/145 (20.0%); I2: 5/35 (14.3%); C: 28/170 (16.5%)  Intra-hepatic cholestasis of pregnancy  I1: 30/145 (20.7%); I2: 5/35 (14.3%); C: 20/170 (11.7%)  *Small for gestational age*  I1: 25/145 (17.2%); I2: 2/35 (5.7%); C: 21/170 (12.3%) |
| Algeri P et al (2018) [21] | Italy | Retrospective cohort study;  Majority of the women in the normal prepregnancy BMI range (~80%); Patients with BMI < 18.5 were excluded | Based on Institute of Medicine (IOM) guidelines  Normal BMI: 1.0 lb./week  Overweight: 0.84 lb./week  Obese: 0.68 lb./week | Adequate GWG (C) vs. low GWG (I 1) and excessive GWG (I2) | *Preterm birth (<37 weeks)*  I1: 61/91 (67.0%); I2: 9/11 (81.8%); C: 29/73 (39.7%)  *Very preterm birth*  I1: 23/91 (25.3%); I2: 3/11 (27.3%); C: 5/73 (6.8%)  *Small for gestational age (SGA)*  I1: 20/91 (22.0%); I2: 0/11 (0.0%); C: 14/73 (19.2%)  *Gestational hypertension or pre-eclampsia*  I1: 7/91 (7.7%); I2: 7/11 (63.6%); C: 13/73 (17.8%)  *Gestational diabetes mellitus*  I1: 15/91 (16.5%); I2: 0/11 (0.0%); C: 7/73 (9.6%)  *“Any” adverse outcomes in any of the twins*  I1: 40/91 (44.0%); I2: 4/11 (36.4%); C: 14/73 (19.2%) |
| Shamshirsaz AA et al (2014) [22] | USA | Multicenter retrospective cohort study; women with different pre-pregnancy BMI were included in the analysis   - Normal weight (BMI 18.5–24.9) - Overweight (BMI 25–29.9) - Obese (BMI≥30)   Those with a pre-pregnancy BMI less than 18.5 kg/m2 were excluded | For normal pre-pregnancy women: 0.46–0.68 kg per week (17–25 kg over 37 weeks)    Pre-pregnancy overweight women: 0.38–0.62 kg per week (14–23 kg over 37 weeks)  Pre-pregnancy obese women: 0.30–0.51 kg per week (11–19 kg over 37 weeks) | Adequate GWG (C) vs. low GWG (I 1) and excessive GWG (I2) | **Among all women**  *Mean (SD) gestational weeks*  I1: 32.8 (3.9) (N=179); I2: 33.5 (3.7) (N=134); C: 34.2 (3.3) (N=257)  *Smaller twin mean (SD) birth weight (g)*  I1: 1716.7 (680) (N=179); I2: 1945.9 (675.3) (N=134); C: 2002.7 (622.3) (N=257)  *Both twins >2500 g (n %)*  I1: 23 (12.8%) (N=179); I2: 30 (22.4%) (N=134); C: 59 (22.9%) (N=257)  *Any twin with IUGR (n%)*  I1: 32 (17.9%) (N=179); I2: 17 (12.7%) (N=134); C: 20 (7.8%) (N=257)  *Gestational hypertension, pre-eclampsia or HELLP syndrome*  I1: 16 (8.9%) (N=179); I2: 28 (20.9%) (N=134); C: 44 (17.1%) (N=257)  *Preterm premature rupture of membrane (n%)*  I1: 55 (30.7%) (N=179); I2: 41 (30.6%) (N=134); C: 76 (29.6%) (N=257)  *Preterm birth (<37 weeks)*  I1: 150 (83.8%) (N=179); I2: 114 (85.1%) (N=134); C: 201 (78.2%) (N=257)  *Very preterm birth (<32 weeks)*  I1: 67 (37.4%) (N=179); I2: 29 (21.6%) (N=134); C: 53 (20.6%) (N=257)  **Among those with pre-pregnancy BMI (18.5-24.9) i.e., normal**  *Mean (SD) gestational weeks*  I1: 32.6 (3.9) (N=107); I2: 33.2 (3.7) (N=60); C: 34.5 (3.3) (N=119)  *Smaller twin mean (SD) birth weight (g)*  I1: 1652 (655) (N=107); I2: 1923 (650) (N=60); C: 2020 (616) (N=119)  *Both twins >2500 g (n %)*  I1: 11 (10.3%) (N=107); I2: 11 (18.3%) (N=60); C: 30 (25.2%) (N=119)  *Any twin with IUGR (n%)*  I1: 28 (13%) (N=107); I2: 6 (5.0%) (N=60); C: 10 (8.4%) (N=119)  *Gestational hypertension, pre-eclampsia or HELLP syndrome*  I1: 8 (7.5%) (N=107); I2: 12 (20.0%) (N=60); C: 20 (16.8%) (N=119)  *Preterm premature rupture of membrane (n%)*  I1: 32 (29.9%) (N=107); I2: 17 (28.3%) (N=60); C: 31 (26.1%) (N=119)  *Preterm birth (<37 weeks)*  I1: 91 (85.1%) (N=107); I2: 50 (83.3%) (N=60); C: 88 (74.0%) (N=119)  *Very preterm birth (<32 weeks)*  I1: 44 (41.1%) (N=107); I2: 14 (23.3%) (N=60); C: 21 (17.7%) (N=119)  **Among those with pre-pregnancy BMI (25–29.9) i.e., overweight**  *Mean (SD) gestational weeks*  I1: 32.6 (4.1) (N=40); I2: 33.7 (3.7) (N=42); C: 33.9 (3.1) (N=79)  *Smaller twin mean (SD) birth weight (g)*  I1: 1716 (694) (N=40); I2: 1939 (664) (N=42); C: 1972 (608) (N=79)  *Both twins >2500 g (n %)*  I1: 6 (15.0%) (N=40); I2: 10 (23.8%) (N=42); C: 15 (19.0%) (N=79)  *Any twin with IUGR (n%)*  I1: 4 (5%) (N=40); I2: 6 (7.3%) (N=42); C: 6 (3.8%) (N=79)  *Gestational hypertension, pre-eclampsia or HELLP syndrome*  I1: 4 (10.0%) (N=40); I2: 7 (16.7%) (N=42); C: 10 (12.7%) (N=79)  *Preterm premature rupture of membrane (n%)*  I1: 13 (32.5%) (N=40); I2: 14 (33.3%) (N=42); C: 30 (38.0%) (N=79)  *Preterm birth (<37 weeks)*  I1: 35 (87.5%) (N=40); I2: 36 (85.7%) (N=42); C: 66 (83.5%) (N=79)  *Very preterm birth (<32 weeks)*  I1: 17 (42.5%) (N=40); I2: 11 (26.2%) (N=42); C: 19 (24.1%) (N=79)  **Among those with pre-pregnancy BMI (BMI≥30) i.e., Obese**  *Mean (SD) gestational weeks*  I1: 33.6 (3.9) (N=32); I2: 33.7 (3.6) (N=32); C: 33.9 (3.4) (N=59)  *Smaller twin mean (SD) birth weight (g)*  I1: 1934 (691) (N=32); I2: 1998 (712) (N=32); C: 2009 (643) (N=59)  *Both twins >2500 g (n %)*  I1: 6 (18.8%) (N=32); I2: 9 (28.1%) (N=32); C: 14 (23.7%) (N=59)  *Any twin with IUGR (n%)*  I1: 0 (0.0%) (N=32); I2: 5 (7.9%) (N=32); C: 4 (3.4%) (N=59)  *Gestational hypertension, pre-eclampsia or HELLP syndrome*  I1: 4 (12.5%) (N=32); I2: 9 (28.1%) (N=32); C: 14 (23.7%) (N=59)  *Preterm premature rupture of membrane (n%)*  I1: 10 (31.3%) (N=32); I2: 10 (31.3%) (N=32); C: 15 (25.4%) (N=59)  *Preterm birth (<37 weeks)*  I1: 24 (75.0%) (N=32); I2: 28 (87.5%) (N=32); C: 47 (79.7%) (N=59)  *Very preterm birth (<32 weeks)*  I1: 6 (18.8%) (N=32); I2: 4 (12.5%) (N=32); C: 13 (22.0%) (N=59) |
| Ozcan T et al (2016) [23] | USA | Retrospective cohort study using data from  the Finger Lakes Region Perinatal Data System (FLRPDS)  and Central New York Region Perinatal Data System  (CNYRPDS); women with different pre-pregnancy BMI were included in the analysis   - Normal weight (BMI 18.5–24.9) - Overweight (BMI 25–29.9) - Obese (BMI≥30)   Those with a pre-pregnancy BMI less than 18.5 kg/m2 were excluded | Normal pre-pregnancy: weekly weight gain of 1–1.4 lbs/week  Overweight women: weekly weight gain of 0.84–1.35 lbs/week  Obese women: weekly weight gain of 0.68–1.13 lbs/week  Weekly weight gain was calculated by dividing total weight gain by week of delivery | Adequate GWG vs. low GWG and excessive GWG | **Among those with pre-pregnancy BMI (18.5-24.9) i.e., normal**  Comparison with normal (reference) weekly weight gain  *Gestational diabetes*  Low: OR 1.27 (95% CI: 0.95, 1.72)  Excessive: OR 1.06 (95% CI: 0.76, 1.46)  *Gestational hypertension, pre-eclampsia or HELLP syndrome*  Low: OR 0.70 (95% CI: 0.55, 0.90)  Excessive: OR 1.97 (95% CI: 1.60, 2.43)  *Preterm delivery (<37 weeks)*  *Low:* OR 1.46 (95% CI: 1.25, 1.70)  Excessive: OR 1.07 (95% CI: 0.90, 1.26)  *Very preterm delivery (<32 weeks)*  Low: OR 1.99 (95% CI: 1.57, 2.51)  Excessive: OR 0.79 (95% CI: 0.58, 1.06)  *Birth weight of one twin <10%*  Low: OR 1.43 (95% CI: 1.19, 1.71)  Excessive: OR 0.64 (95% CI: 0.51, 0.81)  *Birth weight of both twins <10%*  Low: OR 2.09 (95% CI: 1.30, 3.37)  Excessive: OR 0.75 (95% CI: 0.39, 1.44)  **Among those with pre-pregnancy BMI (25–29.9) i.e., overweight**  Comparison with normal (reference) weekly weight gain  *Gestational diabetes*  Low: OR 1.16 (95% CI: 0.83, 1.63)  Excessive: OR 0.67 (95% CI: 0.45, 1.00)  *Gestational hypertension, pre-eclampsia, or HELLP syndrome*  Low: OR 0.81 (95% CI: 0.60, 1.09)  Excessive: OR 1.69 (95% CI: 1.30, 2.18)  *Preterm delivery (<37 weeks)*  *Low:* OR 1.88 (95% CI: 1.54, 2.29)  Excessive: OR 0.94 (95% CI: 0.76, 1.17)  *Very preterm delivery (<32 weeks)*  Low: OR 2.03 (95% CI: 1.51, 2.73)  Excessive: OR 0.88 (95% CI: 0.62, 1.26)  *Birth weight of one twin <10%*  Low: OR 1.91 (95% CI: 1.48, 2.45)  Excessive: OR 0.75 (95% CI: 0.55, 1.02)  *Birth weight of both twins <10%*  Low: OR 2.31 (95% CI: 0.99, 5.39)  Excessive: OR 0.39 (95% CI: 0.10, 1.54)  **Among those with pre-pregnancy BMI (BMI≥30) i.e., Obese**  Comparison with normal (reference) weekly weight gain  *Gestational diabetes*  Low: OR 1.56 (95% CI: 1.22, 1.99)  Excessive: OR 0.89 (95% CI: 0.67, 1.19)  *Gestational hypertension, pre-eclampsia or HELLP syndrome*  Low: OR 0.82 (95% CI: 0.65, 1.05)  Excessive: OR 1.58 (95% CI: 1.26, 1.99)  *Preterm delivery (<37 weeks)*  *Low:* OR 1.36 (95% CI: 1.13, 1.64)  Excessive: OR 1.22 (95% CI: 0.99, 1.49)  *Very preterm delivery (<32 weeks)*  Low: OR 1.99 (95% CI: 1.49, 2.68)  Excessive: OR 0.92 (95% CI: 0.64, 1.32)  *Birth weight of one twin <10%*  Low: OR 1.63 (95% CI: 1.30, 2.05)  Excessive: OR 0.81 (95% CI: 0.61, 1.08)  *Birth weight of both twins <10%*  Low: OR 6.68 (95% CI: 2.63, 16.9)  Excessive: OR 0.52 (95% CI: 0.10, 2.65) |
| Lutsiv O et al (2017) [24] | Canada | Retrospective cohort study of all women  who gave birth to twins between January 1, 2003 and  December 31, 2014 in Nova Scotia; Those with a pre-pregnancy BMI less than 18.5 kg/m2 ; were excluded; Stratified analysis based on pre-pregnancy maternal BMI was not done | The total GWG recommendations for twin pregnancies  for normal weight, overweight and obese women  are: 16.8–24.5 kg, 14.1–22.7 kg and 11.4–19.1 kg, respectively | Adequate GWG (C) vs. low GWG (I 1) and excessive GWG (I2) | *Small for gestation age (<10^th^ percentile)*  I1: 121/402 (30.1%); I2: 87/440 (19.8%); C: 135/640 (21.1%)  *Low birth weight (<2500g)*  I1: 204/402 (50.8%); I2: 155/440 (35.2%); C: 244/640 (38.1%)  *Mean (SD) birth weight*  I1: 2475 (110.8) (N=402); I2: 2637 (92.3) (N=440); C: 2653 (102.5) (N=640)  *NICU admission*  I1: 170/402 (42.3%); I2: 155/440 (35.2%); C: 230/640 (35.9%)  *Mean (SD) gestational age in weeks*  I1: 36.9 (0.40) (N=201); I2: 37.0 (0.37) (N=220); C: 37.1 (0.38) (N=320)  *Preterm birth (<37 weeks)*  I1: 104/201 (51.7%); I2: 107/220 (48.6%); C: 142/320 (44.4%)  *Caesarean delivery*  I1: 91/201 (45.3%); I2: 126/220 (57.3%); C: 170/320 (53.1%)  *Postpartum hemorrhage*  I1: 42/201 (20.9%); I2: 43/220 (19.6%); C: 63/320 (19.7%) |
| Lin D et al (2019) [25] | China | Retrospective cohort study of pregnant women; Stratified analysis based on pre-pregnancy maternal BMI was not done | The rates of recommended weight  gain, obtained by dividing the recommended weight gain  by 37 weeks, were : 0.459–0.676 kg per week for  normal weight; 0.378–0.622 kg per week for overweight  and 0.297–0.514 kg per week for obesity women | Adequate GWG (C) vs. low GWG (I 1) and excessive GWG (I2) | *Gestational hypertension, pre-eclampsia, or HELLP syndrome*  I1: 10/97 (10.3%); I2: 82/281 (29.2%); C: 38/267 (14.2%)  *Gestational anemia*  I1: 49/97 (50.5%); I2: 123/281 (43.8%); C: 101/267 (37.8%)  *Premature rupture of membrane*  I1: 12/97 (12.4%); I2: 27/281 (9.6%); C: 37/267 (13.9%)  *Preterm (<37 weeks)*  I1: 75/97 (77.3%); I2: 208/281 (74.0%); C: 189/267 (70.8%)  *Very preterm (<32 weeks)*  I1: 5/97 (5.2%); I2: 10/281 (3.6%); C: 12/267 (4.5%)  *Birth weight <2500 g*  I1: 149/194 (76.8%); I2: 314/562 (55.9%); C: 360/534 (67.4%)  *Small for gestational age*  I1: 16/194 (8.3%); I2: 24/562 (4.3%); C: 29/534 (5.4%)  *NICU admission*  I1: 88/194 (45.4%); I2: 196/562 (34.9%); C: 206/534 (38.6%) |
| Lal AK et al (2015) [26] | USA | Retrospective cohort study | 0.46 to 0.68 kg per week for  normal-weight women, 0.38 to 0.62 kg per week for overweight  women and 0.3 to 0.51 kg per week for obese women | Adequate GWG (C) vs. low GWG (I 1) and excessive GWG (I2) | **Among all women**  *Smaller twin mean (SD) birth weight (g)*  I1: 2123 (566) (N=1040); I2: 2375.1 (504.7) (N=517); C: 2258.8 (562) (N=1097)  *Mean (SD) gestational age in weeks*  I1: 35.0 (3.1) (N=1040); I2: 35.7 (2.4) (N=517); C: 35.5 (2.9) (N=1097)  *NICU admission*  I1: 508/1040 (48.8%); I2: 227/517 (43.9%); C: 474/1097 (43.2%)  *Small for gestational age*  I1: 149/1040 (14.3%); I2: 42/517 (8.1%); C: 125/1097 (11.4%)  *Both twins with birth weight <2500 g*  I1: 595/1040 (57.2%); I2: 183/517 (35.4%); C: 469/1097 (42.7%)  *Gestational diabetes:*  I1: 41/1040 (3.9%); I2: 15/517 (2.9%); C: 46/1097 (4.2%)  *Gestational hypertension, pre-eclampsia, or HELLP syndrome*  I1: 93/1040 (8.9%); I2: 137/517 (26.5%); C: 160/1097 (14.6%)  **Among those with pre-pregnancy BMI (18.5-24.9) i.e., normal**  *Smaller twin mean (SD) birth weight (g)*  I1: 2103 (578) (N=593); I2: 2359 (498) (N=233); C: 2253 (523) (N=671)  *Mean (SD) gestational age in weeks*  I1: 35.1 (3.2) (N=593); I2: 35.7 (2.5) (N=233); C: 35.5 (2.7) (N=671)  *NICU admission*  I1: 286/593 (48.2%); I2: 101/233 (43.5%); C: 282/671 (42.0%)  *Small for gestational age*  I1: 96/593 (16.2%); I2: 21/233 (9.1%); C: 79/671 (11.8%)  *Both twins with birth weight <2500 g*  I1: 340/593 (57.2%); I2: 84/233 (36.2%); C: 296/671 (44.1%)  *Gestational diabetes:*  I1: 14/593 (2.4%); I2: 3/233 (1.3%); C: 19/671 (2.8%)  *Gestational hypertension, pre-eclampsia, or HELLP syndrome*  I1: 51/593 (8.6%); I2: 61/233 (26.2%); C: 88/671 (13.1%)  **Among those with pre-pregnancy BMI (25–29.9) i.e., overweight**  *Smaller twin mean (SD) birth weight (g)*  I1: 2144 (607) (N=222); I2: 2408 (473) (N=137); C: 2276 (601) (N=247)  *Mean (SD) gestational age in weeks*  I1: 34.9 (3.3) (N=222); I2: 35.9 (2.2) (N=137); C: 35.5 (2.9) (N=247)  *NICU admission*  I1: 109/222 (49.1%); I2: 63/137 (46.0%); C: 112/247 (45.3%)  *Small for gestational age*  I1: 29/222 (13.1%); I2: 9/137 (6.6%); C: 30/247 (12.2%)  *Both twins with birth weight <2500 g*  I1: 119/222 (53.6%); I2: 49/137 (35.8%); C: 99/247 (40.1%)  *Gestational diabetes:*  I1: 12/222 (5.4%); I2: 2/137 (1.5%); C: 16/247 (6.5%)  *Gestational hypertension, pre-eclampsia or HELLP syndrome*  I1: 19/222 (8.5%); I2: 30/137 (21.9%); C: 34/247 (13.8%)  **Among those with pre-pregnancy BMI (BMI≥30) i.e., Obese**  *Smaller twin mean (SD) birth weight (g)*  I1: 2155 (513) (N=225); I2: 2370 (543) (N=147); C: 2257 (562) (N=179)  *Mean (SD) gestational age in weeks*  I1: 35.0 (2.8) (N=225); I2: 35.6 (2.6) (N=147); C: 35.3 (3.0) (N=179)  *NICU admission*  I1: 113/225 (50.2%); I2: 63/147 (42.8%); C: 80/179 (44.4%)  *Small for gestational age*  I1: 24/225 (10.7%); I2: 12/147 (8.2%); C: 16/179 (8.9%)  *Both twins with birth weight <2500 g*  I1: 136/225 (60.4%); I2: 50/147 (34.3%); C: 74/179 (41.1%)  *Gestational diabetes:*  I1: 15/225 (6.7%); I2: 10/147 (6.9%); C: 11/179 (6.1%)  *Gestational hypertension, pre-eclampsia, or HELLP syndrome*  I1: 23/225 (10.2%); I2: 46/147 (31.3%); C: 38/179 (21.2%) |
| Gavard JA et al (2014) [27] | USA | Record based cohort study; All obese pregnant women (BMI >=30.0 kg/m2) residing in  Missouri | Three  categories of gestational weight gain were examined: *<*25 pounds (low), 25–42 pounds (adequate),  and*>*42 pounds (excessive). The 25–42 pound group served as the reference category for all comparisons | Adequate GWG (C) vs. low GWG (I 1) and excessive GWG (I2) | *Mean (SD) gestational age in weeks*  I1: 37.0 (1.9) (N=512); I2: 38.0 (2.1) (N=504); C: 38.0 (2.7) (N=646)  *Mean (SD) birth weight (g) for the smaller twin*  I1: 2580 (313) (N=512); I2: 2715 (289) (N=504); C: 2665 (119) (N=646)  *Any one twin with birth weight <2500 g*  I1: 144/512 (28.1%); I2: 87/504 (17.3%); C: 122/646 (18.9%)  *Gestational hypertension, pre-eclampsia, or HELLP syndrome*  I1: 21/256 (8.2%); I2: 36/252 (14.3%); C: 30/323 (9.3%)  *Cesarean delivery*  I1: 162/256 (63.3%); I2: 179/252 (71.0%); C: 224/323 (69.4%) |
| Bodnar LM et al (2019) [28] | USA | Population based retrospective cohort study; women with different pre-pregnancy BMI were included in the analysis   - Normal weight (BMI 18.5–24.9) - Overweight (BMI 25–29.9)   Obese (BMI≥30) | Authors standardized total gestational weight  gain (kg) for gestational age at delivery using prepregnancy  BMI category-specific z score charts for twin  pregnancies that were produced for Magee-Women’s  Hospital in Pittsburgh, Pennsylvania   - Low weight gain defined as <-1 z score - Adequate weight gain defined as -1 to +1 z score - Excessive weight gain defined as >+1 z score | Adequate GWG (C) vs. low GWG (I 1) and excessive GWG (I2) | **Among all women**  *Small for gestational age*  I1: 1552/8233 (18.8%); I2: 824/8754 (9.4%); C: 4030/35992 (11.2%)  *Very preterm delivery (<32 weeks)*  I1: 1318/8233 (16.0%); I2: 1196/8754 (13.7%); C: 4062/35992 (11.3%)  *Cesarean delivery*  I1: 5298/8233 (64.3%); I2: 6368/8754 (72.7%); C: 24502/35992 (68.1%)  **Among those with pre-pregnancy BMI (18.5-24.9) i.e., normal**  *Small for gestational age*  I1: 872/4194 (21.0%); I2: 402/4303 (9.3%); C: 2049/17514 (12.0%)  *Very preterm delivery (<32 weeks)*  I1: 673/4194 (16.0%); I2: 569/4303 (13%); C: 1941/17514 (11%)  *Cesarean delivery*  I1: 2549/4194 (61.0%); I2: 3003/4303 (70%); C: 11427/17514 (65%)  **Among those with pre-pregnancy BMI (25–29.9) i.e., overweight**  *Small for gestational age*  I1: 335/1856 (18.0%); I2: 225/2297 (9.8%); C: 971/9220 (11.0%)  *Very preterm delivery (<32 weeks)*  I1: 305/1856 (17.0%); I2: 321/2297 (14%); C: 1041/9220 (11.0%)  *Cesarean delivery*  I1: 1226/1856 (66.0%); I2: 1687/2297 (73%); C: 6324/9220 (69.0%)  **Among those with pre-pregnancy BMI (BMI≥30) i.e., Obese**  *Small for gestational age*  I1: 345/2183 (15.8%); I2: 197/2154 (9.2%); C: 1010/9258 (10.9%)  *Very preterm delivery (<32 weeks)*  I1: 340/2183 (15.6%); I2: 306/2154 (14.2%); C: 1080/9258 (11.7%)  *Cesarean delivery*  I1: 1523/2183 (69.8%); I2: 1678/2154 (77.9%); C: 6751/9258 (72.9%) |
| Pecheux O et al (2019) [29] | France | Retrospective analysis of cohort data; all twin gestations with two live born infants delivered after 23 weeks and 6 days of amenorrhea; Stratified analysis based on pre-pregnancy maternal BMI was not done | 0.46 to 0.68 kg per week for  normal-weight women, 0.38 to 0.62 kg per week for overweight  women and 0.3 to 0.51 kg per week for obese women | Adequate GWG (C) vs. low GWG (I 1) and excessive GWG (I2) | *Gestational hypertension, pre-eclampsia, or HELLP syndrome*  I1: 25/468 (5.3%); I2: 19/64 (29.7%); C: 47/346 (13.6%)  *Premature rupture of membrane*  I I1: 95/468 (20.5%); I2: 12/64 (18.8%); C: 61/346 (17.7%)  *Preterm (<37 weeks)*  I I1: 126/468 (26.9%); I2: 15/64 (23.4%); C: 60/346 (17.3%)  *Very preterm (<32 weeks)*  I I1: 48/468 (10.3%); I2: 6/64 (9.4%); C: 18/346 (5.2%)  *Gestational diabetes mellitus*  I I1: 151/468 (36.9%); I2: 22/64 (39.3%); C: 94/346 (30.6%)  *Postpartum hemorrhage*  I I1: 39/468 (9.2%); I2: 10/64 (17.2%); C: 35/346 (11.2%)  *Cesarean delivery*  I I1: 149/468 (31.8%); I2: 22/64 (34.4%); C: 108/346 (31.3%)  *Birth weight <2500 g*  I I1: 362/468 (77.5%); I2: 40/64 (62.5%); C: 222/346 (64.2%)  *Small for gestational age*  I I1: 235/468 (50.8%); I2: 22/64 (34.4%); C: 165/346 (48.3%)  *NICU admission*  I I1: 148/468 (31.6%); I2: 20/64 (31.3%); C: 84/346 (24.3%) |
