# Supplementary material for: Gestational Weight Gain and Its Effects on Maternal and Neonatal Outcome in Women With Twin Pregnancies: A Systematic Review and Meta-Analysis
Source: Front Pediatr. 2021 Jul 9;9:674414. doi: 10.3389/fped.2021.674414 (PMC8298912; doi:10.3389/fped.2021.674414)
Supplement: Supplementary file 2 [file Table_2.docx]

**Supplementary table 2. Study quality, as assessed using the adapted Ottawa-Newcastle Risk of Bias Assessment tool**

|  | Pettit KE et al (2015) | Wang L et al (2018) | Algeri P et al (2018) | Shamshirsaz AA et al (2014) | Ozcan T et al (2016) | Lutsiv O et al (2017) | Lin D et al (2019) | Lal AK et al (2015) | Gavard JA et al (2014) | Bodnar LM et al (2019) | Pecheux O et al (2019) |
| --- | --- | --- | --- | --- | --- | --- | --- | --- | --- | --- | --- |
| Representativeness/appropriateness of participant selection  Random or consecutive recruitment=Y  Convenience sample=N  Not reported or unclear | Y | Y | Y | Y | Y | Y | Y | Y | Y | Y | Y |
| Control for baseline differences in cohorts  Similarity of groups at baseline or adjustment in analyses=Y  No attempt to control or adjust=N  Not reported=NR | Y | Y | Y | Y | Y | Y | Y | Y | Y | Y | Y |
| Loss to follow-up  Explanation provided for loss of participants and/or intention to treat=Y  No explanation =N | Y | Y | N | Y | N | N | Y | Y | Y | Y | Y |
| Masking of exposure to outcomes assessor  Description of masking=Y  No masking or no description =N | Y | Y | Y | Y | Y | Y | Y | Y | Y | Y | Y |
| Ascertainment of condition  Description of ascertainment/diagnostic criteria=Y  No description or patient self-report=N | Y | Y | Y | Y | Y | N | Y | Y | Y | Y | Y |
| Documentation of other treatment modalities  Documentation=Y  No documentation=N | Y | Y | Y | Y | Y | Y | Y | Y | Y | Y | Y |
| Extent to which valid outcomes are described  Adequate description of outcome=Y  Insufficient detail regarding outcome or follow-up time=N | Y | Y | Y | Y | N | Y | Y | N | Y | Y | Y |
| Prespecification of harms, mode of harms collection  Description of a list of harms assessed or monitoring=Y  No such description or passive harms collection=N  No adverse events reported=NA | Y | N | Y | N | Y | N | Y | N | Y | Y | Y |
| Financial Conflict of interest (COI)  Funding source reported=Y  Funding source not reported=N | Y | Y | Y | N | Y | Y | Y | Y | Y | Y | Y |
