# Supplementary material for: Gestational Weight Gain and Its Effects on Maternal and Neonatal Outcome in Women With Twin Pregnancies: A Systematic Review and Meta-Analysis
Source: Front Pediatr. 2021 Jul 9;9:674414. doi: 10.3389/fped.2021.674414 (PMC8298912; doi:10.3389/fped.2021.674414)
Supplement: Supplementary file 3 [file Table_3.docx]

**Supplementary table 1. Search strategy for inclusion candidate studies**

| **Search strategy**  #1 (pregnancy weight gain OR gestational weight gain)  #2 (pregnancy outcomes OR maternal outcome OR neonatal outcome OR perinatal outcome OR delivery outcome OR adverse birth outcomes)  #3 (twin pregnancy OR dichorionic twin pregnancy OR monochorionic twin pregnancy)  #4 (#1 AND #2 AND #3) |
| --- |
