# Supplementary material for: Gestational Weight Gain and Its Effects on Maternal and Neonatal Outcome in Women With Twin Pregnancies: A Systematic Review and Meta-Analysis
Source: Front Pediatr. 2021 Jul 9;9:674414. doi: 10.3389/fped.2021.674414 (PMC8298912; doi:10.3389/fped.2021.674414)

Supplementary Figure 1. Funnel plot for publication bias when comparing inadequate/low weight gain and adequate weight gain with respect to maternal outcomes

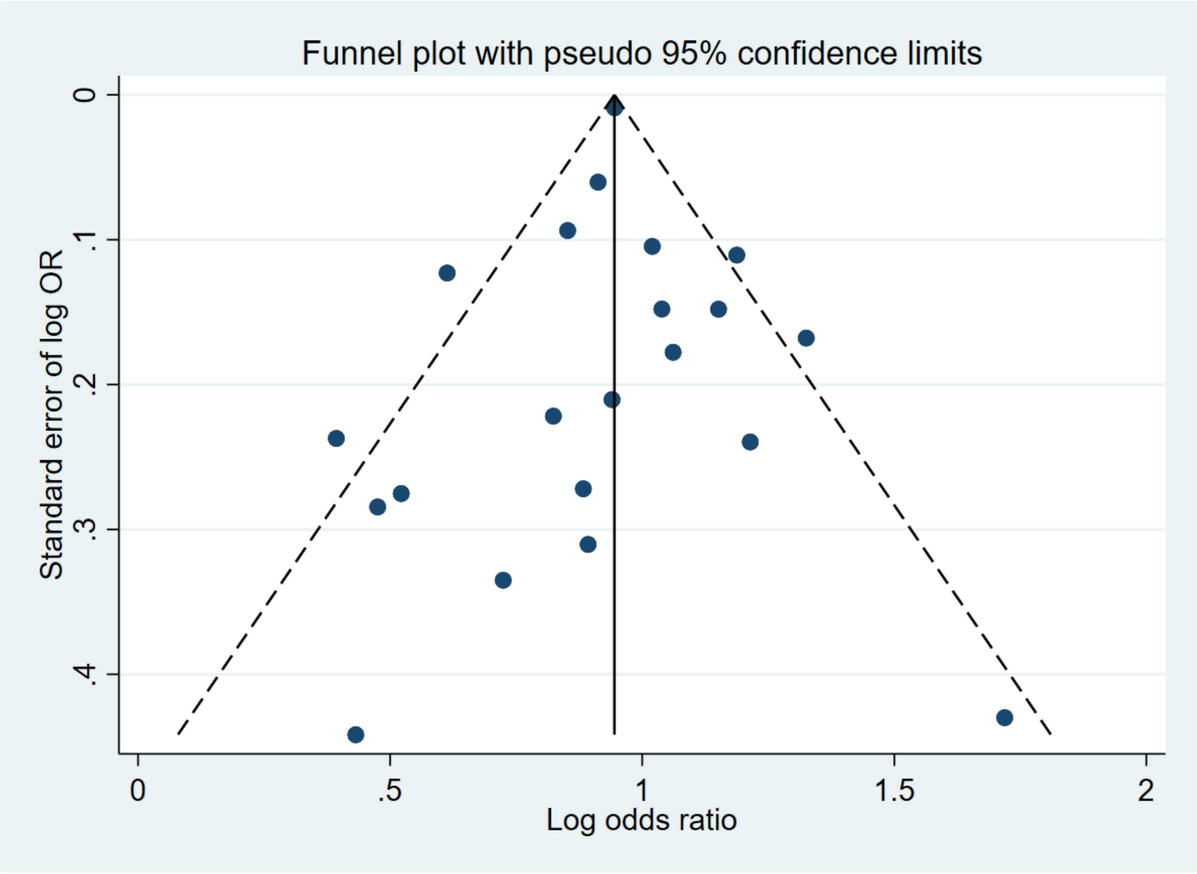

Supplementary Figure 2. Funnel plot for publication bias when comparing inadequate/low weight gain and adequate weight gain with respect to neonatal outcomes

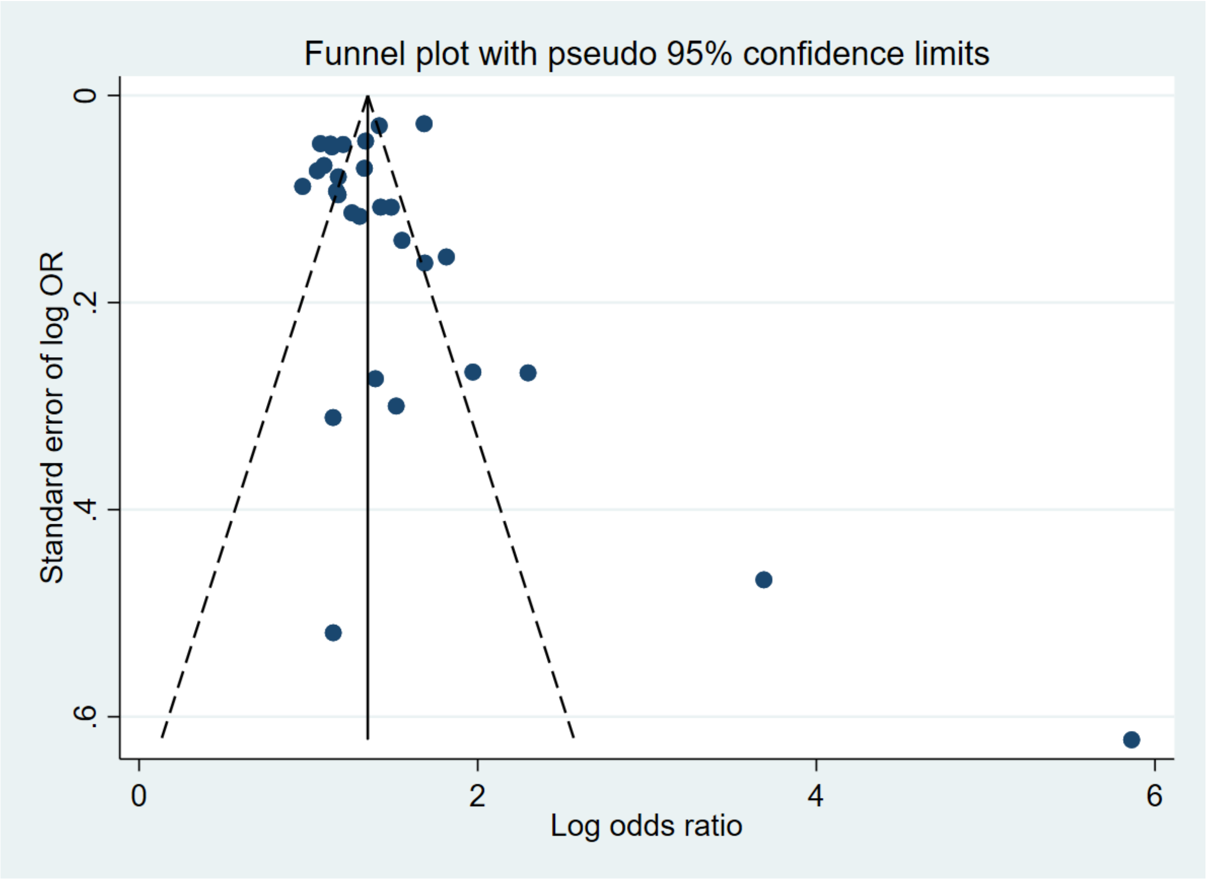

Supplementary Figure 3. Funnel plot for publication bias when comparing excessive weight gain and adequate weight gain with respect to maternal outcomes

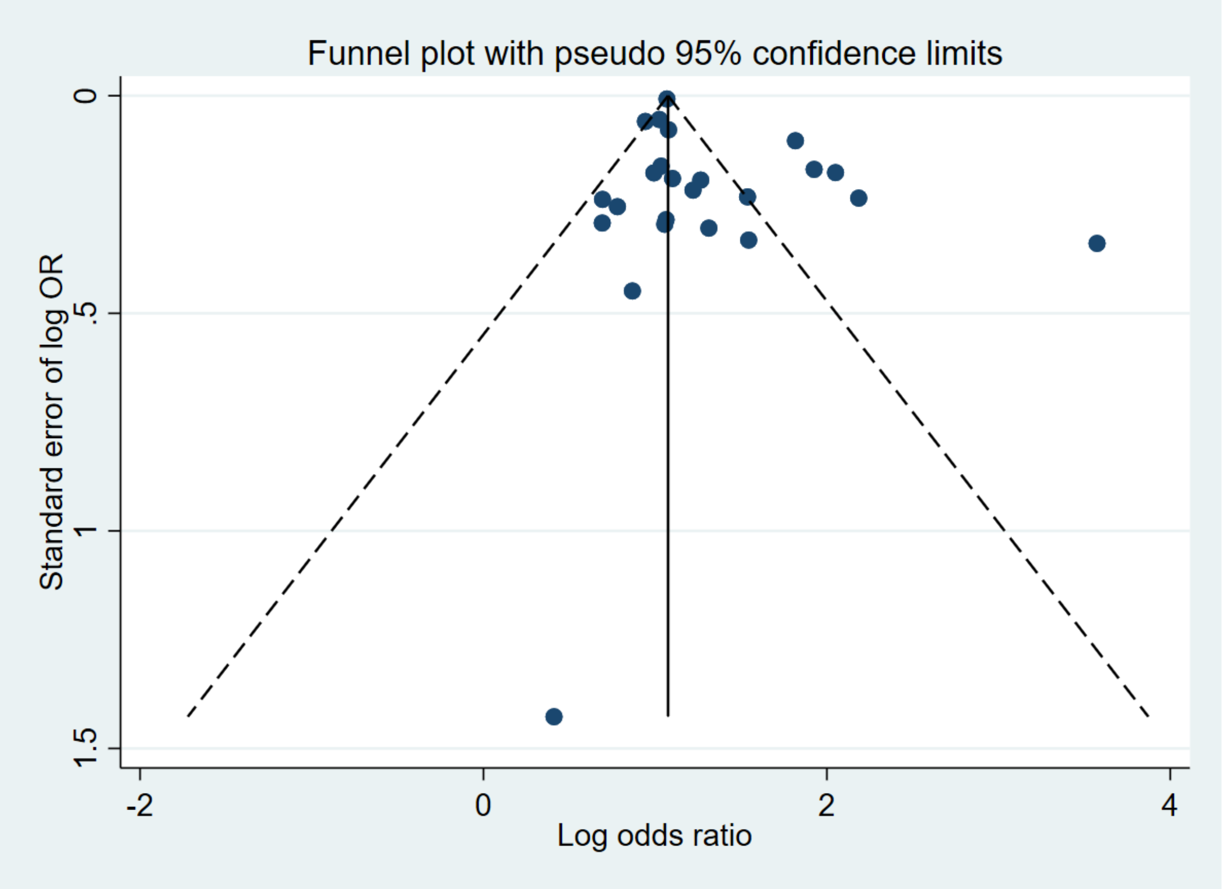

Supplementary Figure 4. Funnel plot for publication bias when comparing excessive weight gain and adequate weight gain with respect to neonatal outcomes

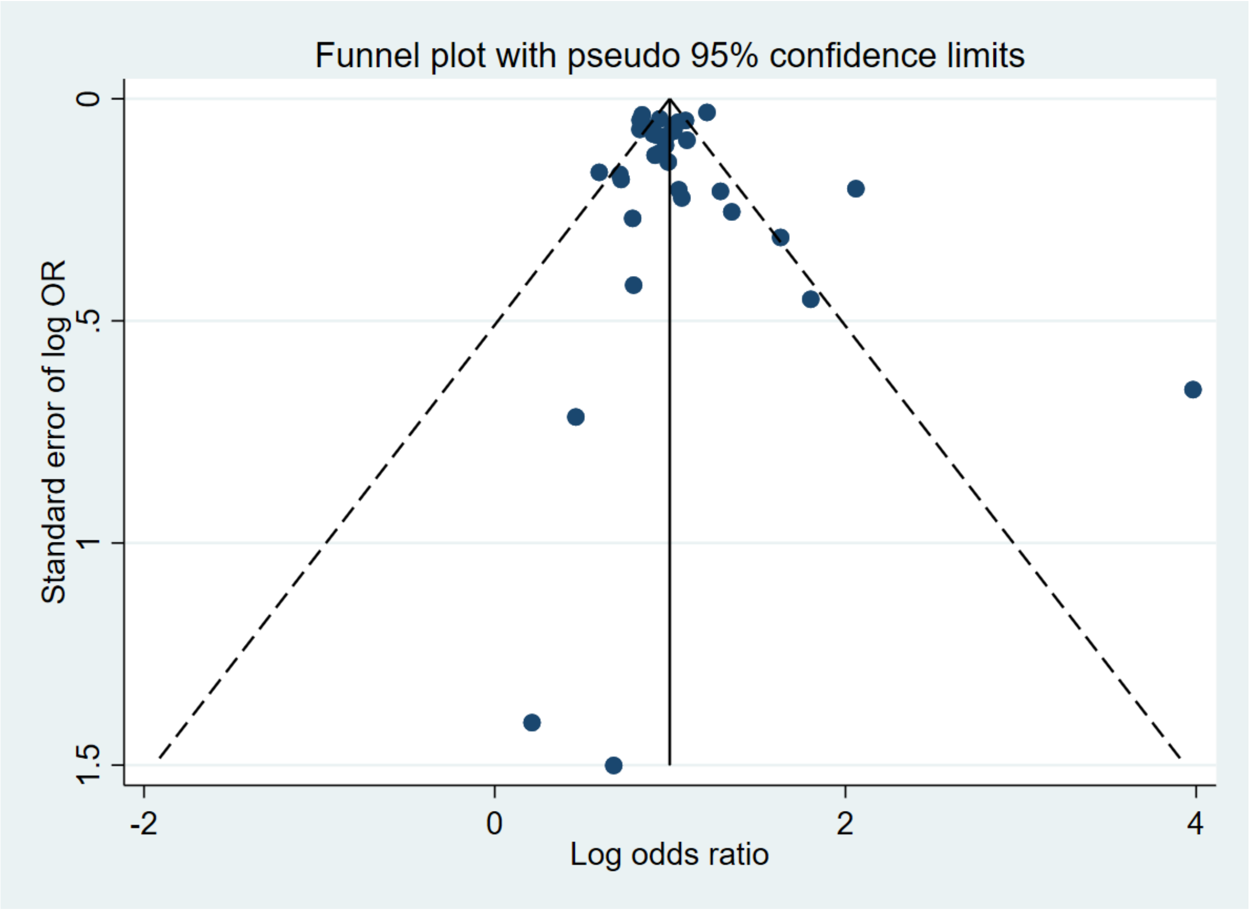

Supplementary Figure 5. Forest plot for neonatal outcomes in mothers with normal pre-pregnancy BMI (inadequate/low weight gain vs. adequate weight gain)

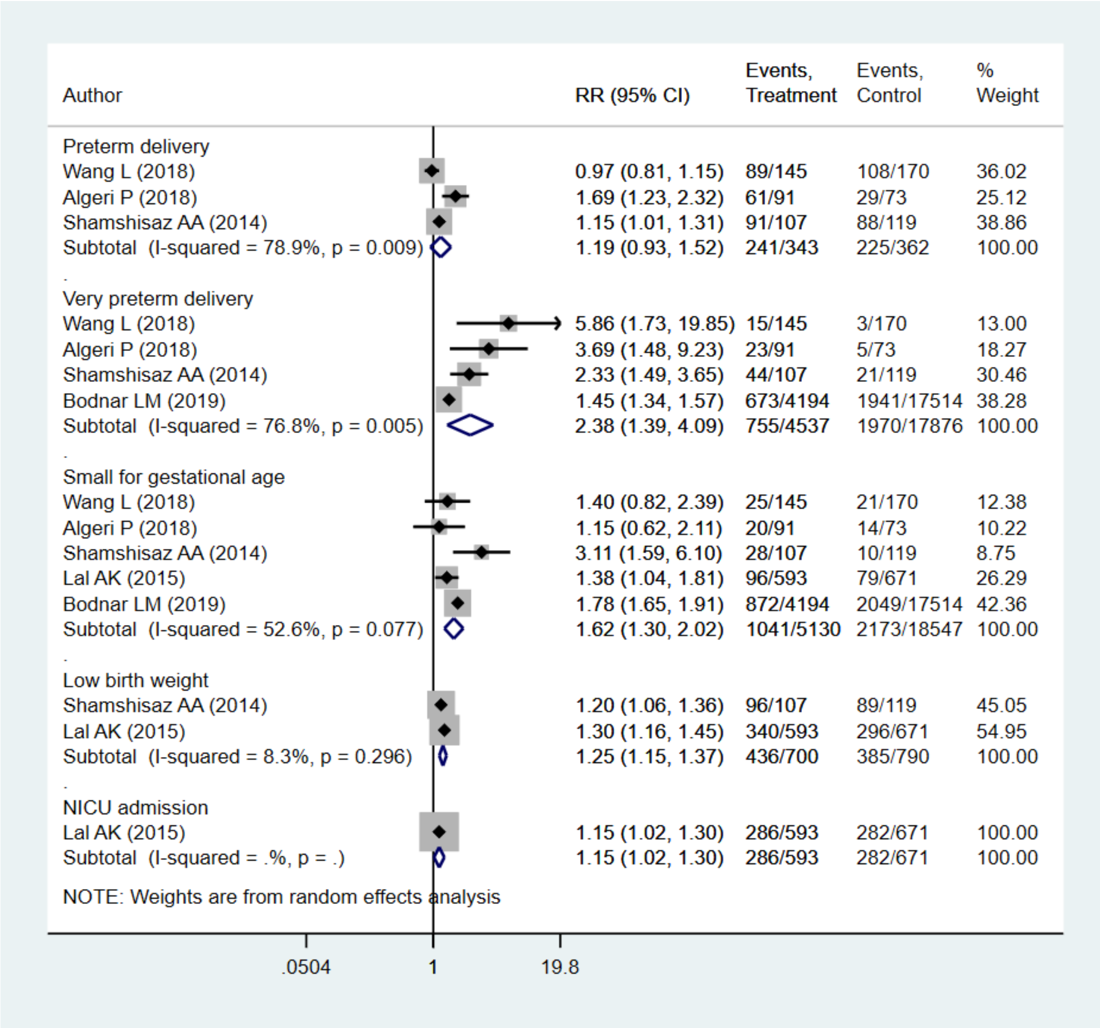

Supplementary Figure 6. Forest plot for maternal outcomes in mothers with normal pre-pregnancy BMI (inadequate/low weight gain vs. adequate weight gain)

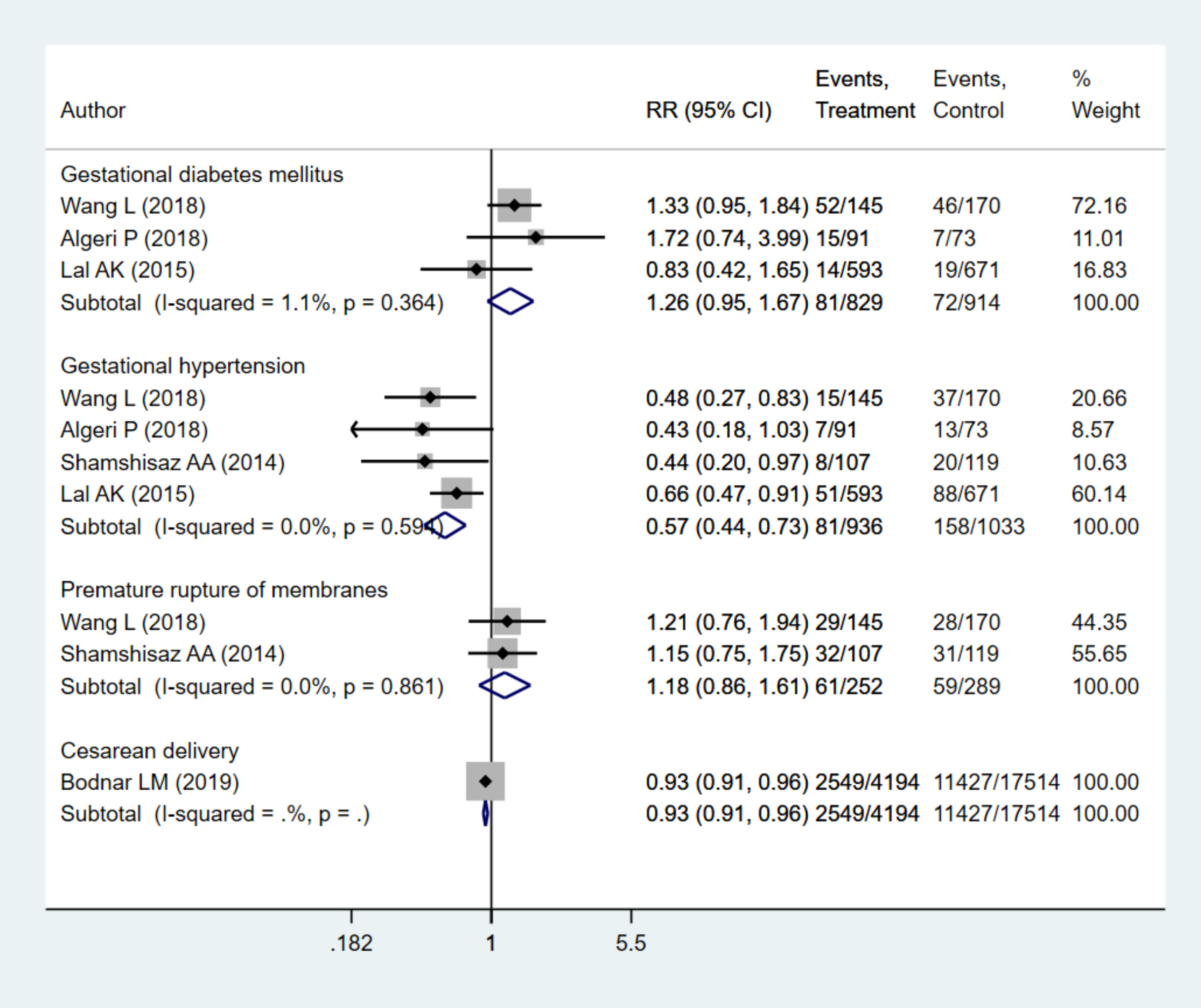

Supplementary Figure 7. Forest plot for maternal outcomes in mothers with normal pre-pregnancy BMI (excessive weight gain vs. adequate weight gain)

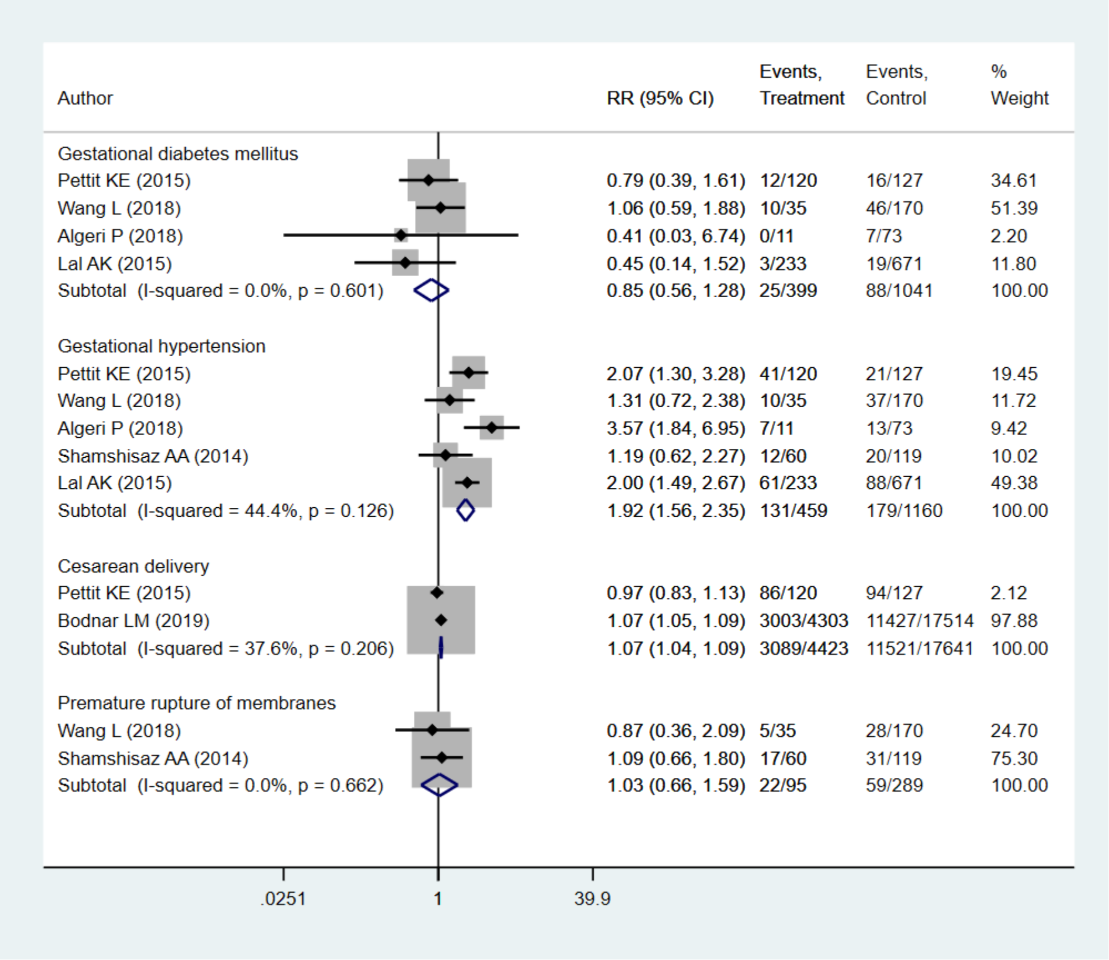

Supplementary Figure 8. Forest plot for neonatal outcomes in mothers with normal pre-pregnancy BMI (excessive weight gain vs. adequate weight gain)

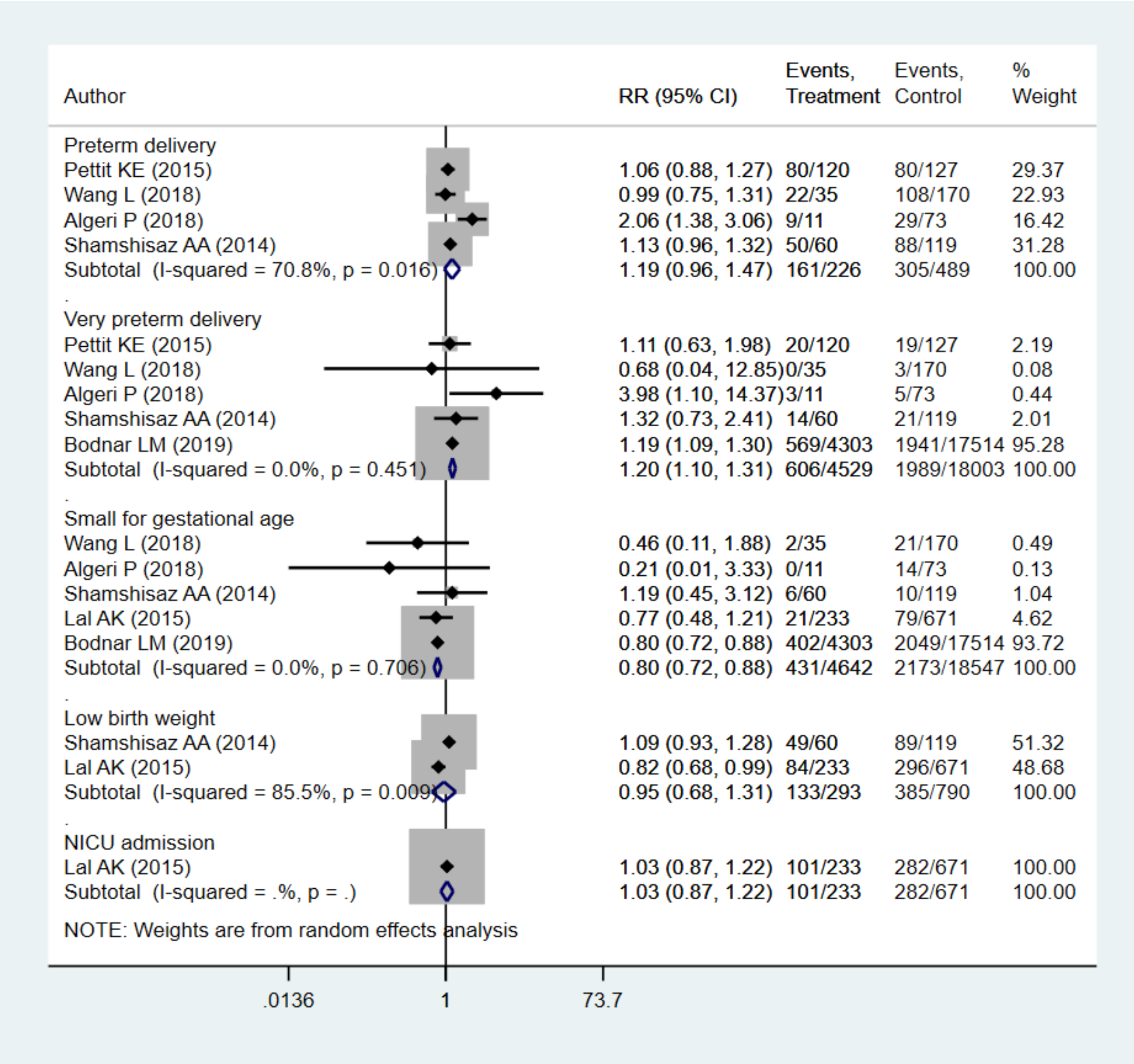

Supplementary Figure 9. Forest plot for neonatal outcomes in overweight mothers (low/inadequate weight gain vs. adequate weight gain)

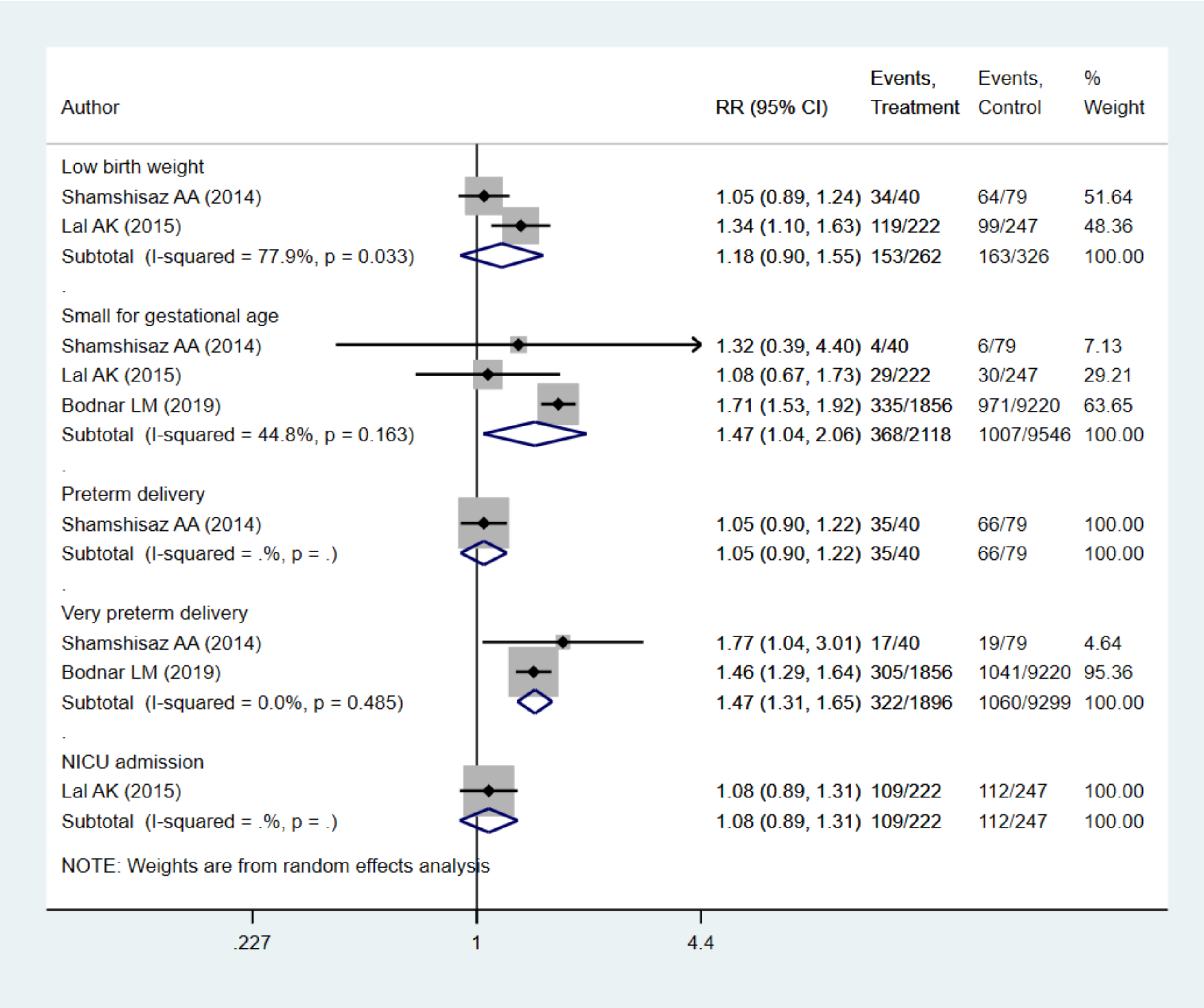

Supplementary Figure 10. Forest plot for maternal outcomes in overweight mothers (low/inadequate weight gain vs. adequate weight gain)

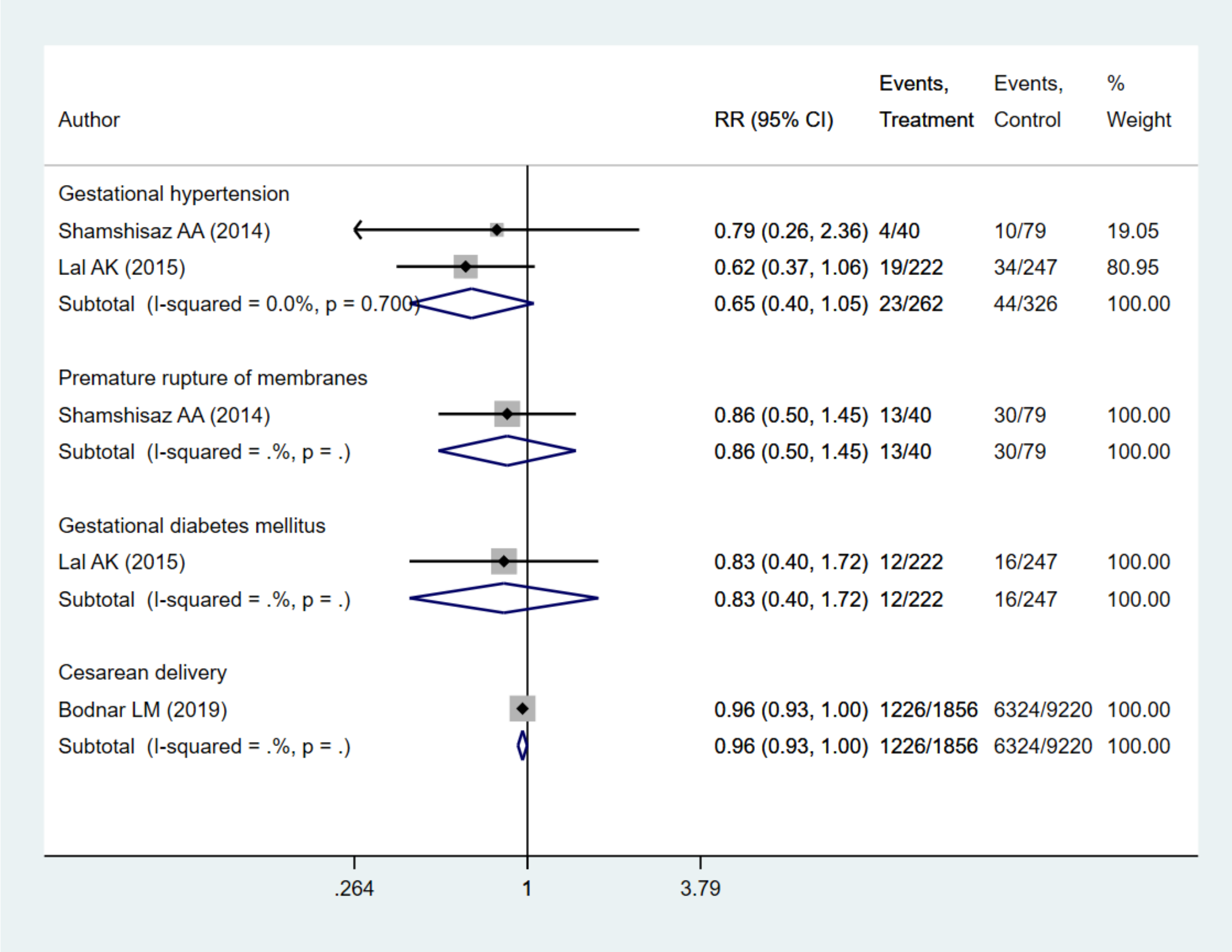

Supplementary Figure 11. Forest plot for maternal outcomes in overweight mothers (excessive weight gain vs. adequate weight gain)

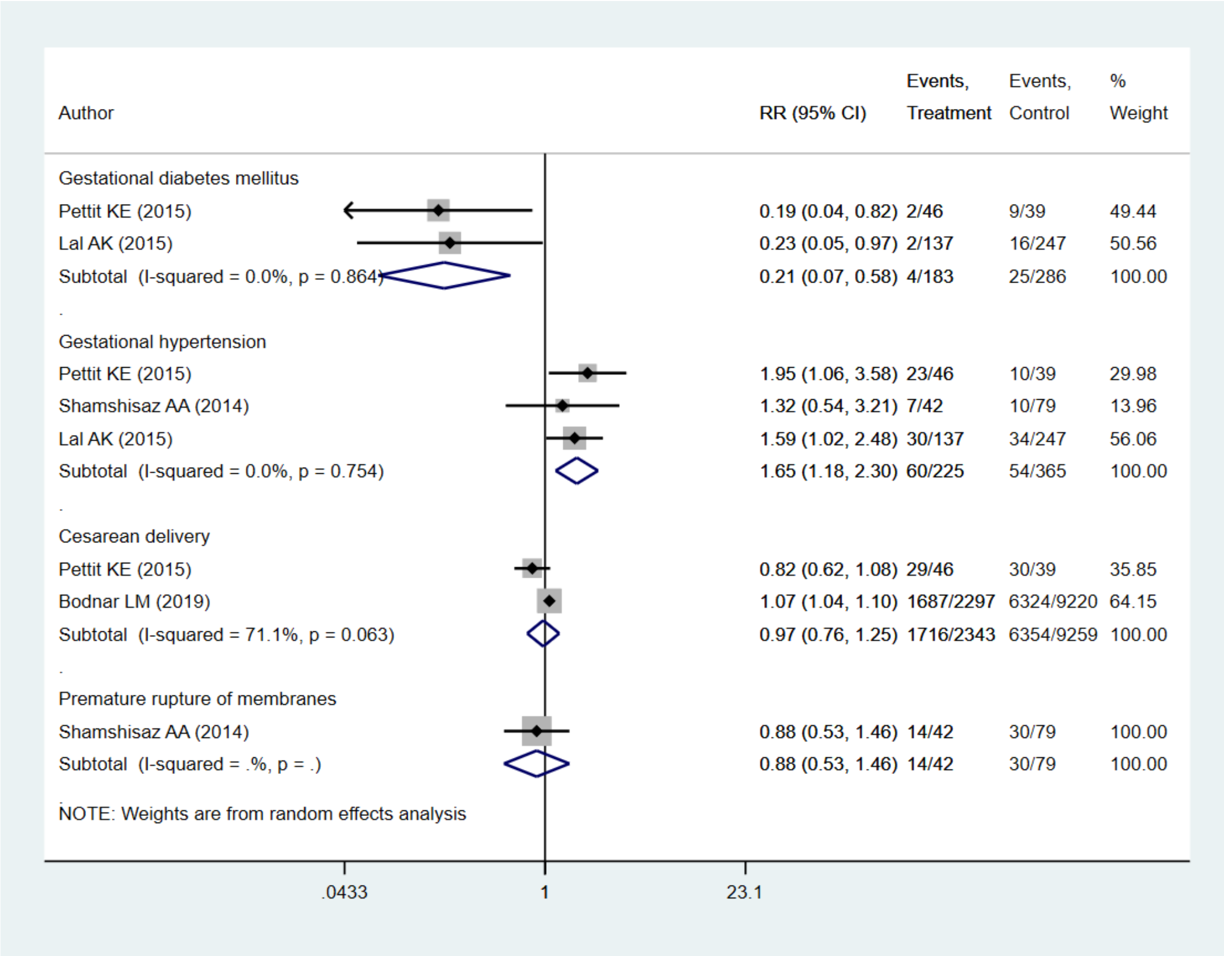

Supplementary Figure 12. Forest plot for neonatal outcomes in overweight mothers (excessive weight gain vs. adequate weight gain)

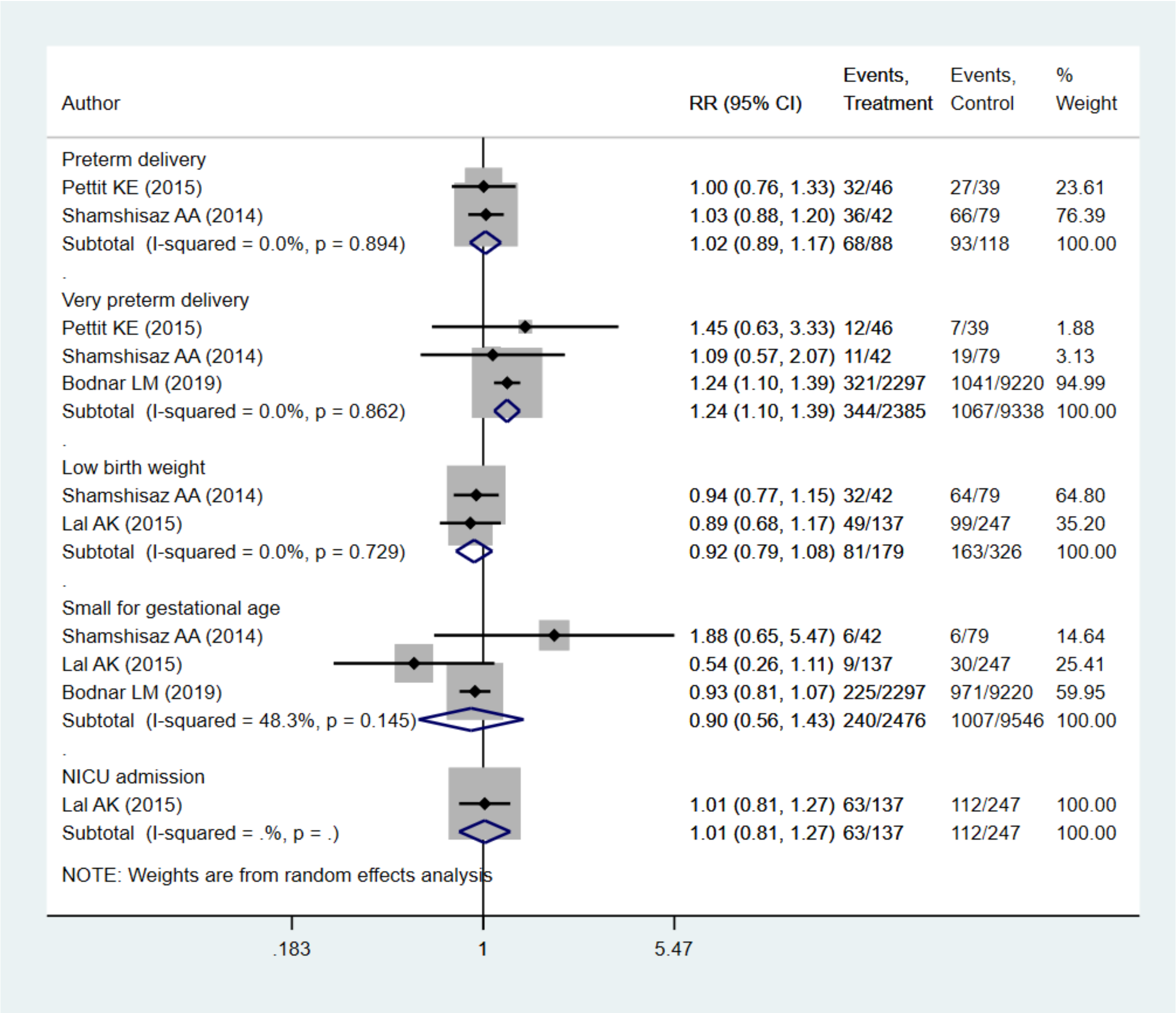

Supplementary Figure 13. Forest plot for neonatal outcomes in obese mothers (inadequate/low weight gain vs. adequate weight gain)

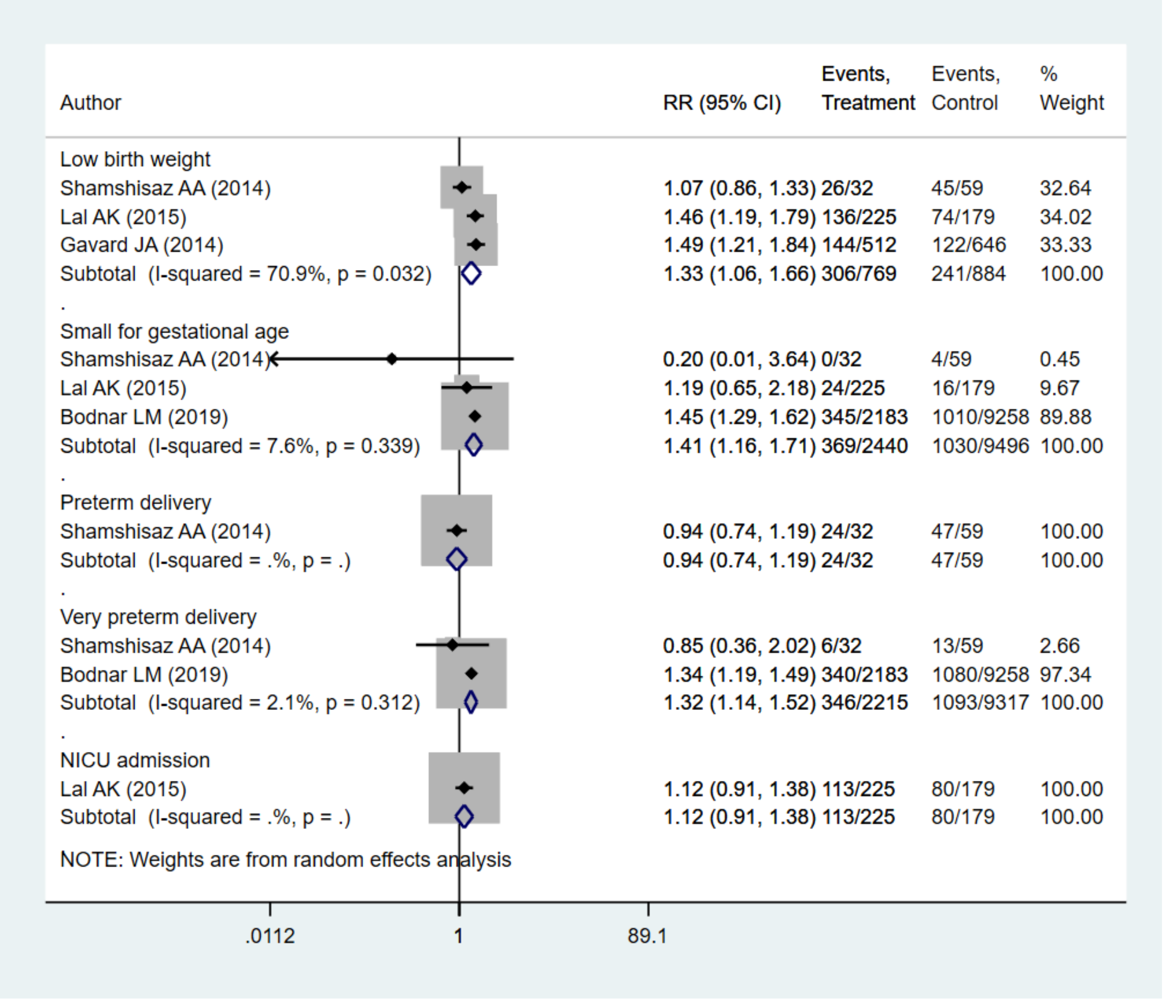

Supplementary Figure 14. Forest plot for maternal outcomes in obese mothers (inadequate/low weight gain vs. adequate weight gain)

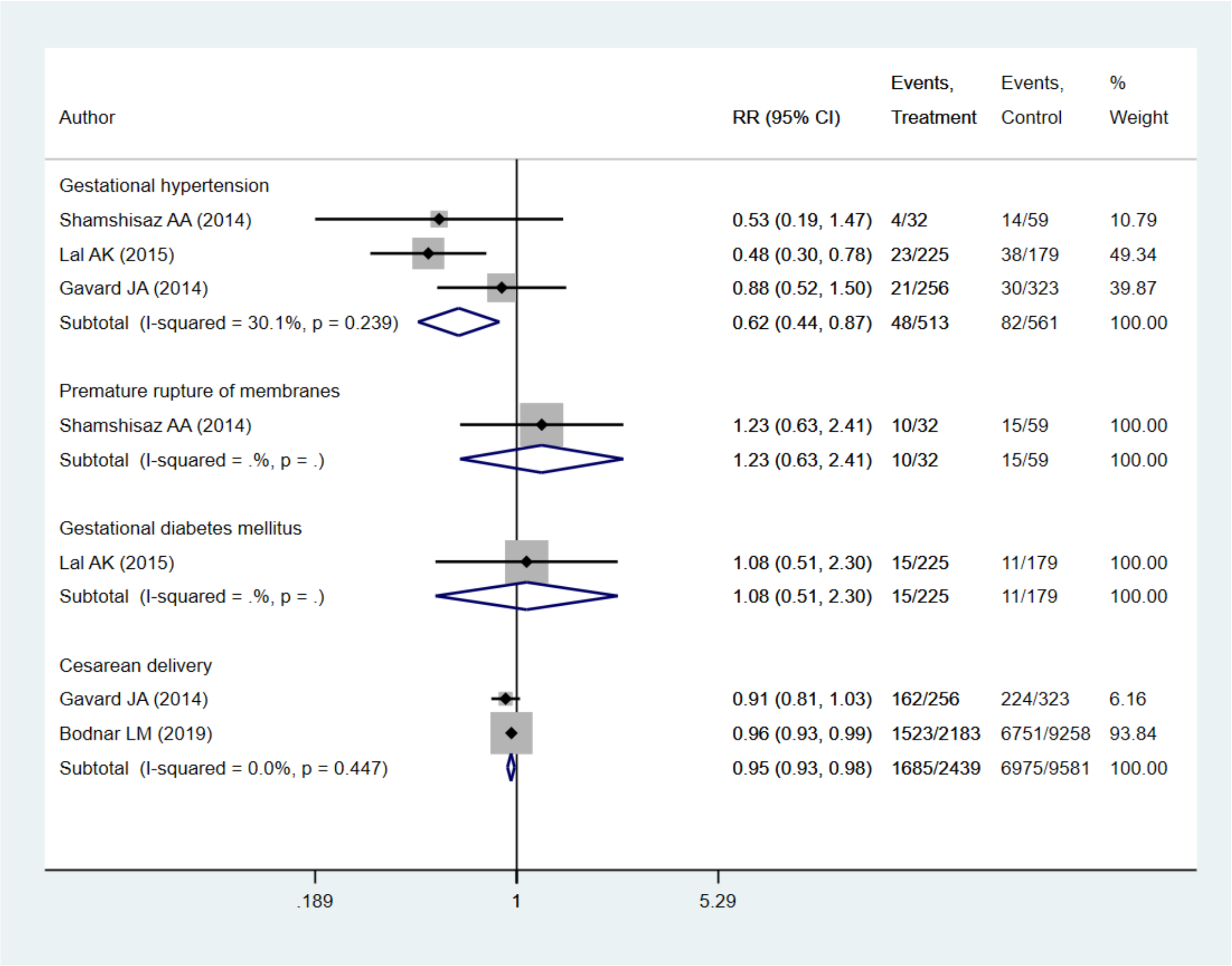

Supplementary Figure 15. Forest plot for maternal outcomes in obese mothers (excessive weight gain vs. adequate weight gain)

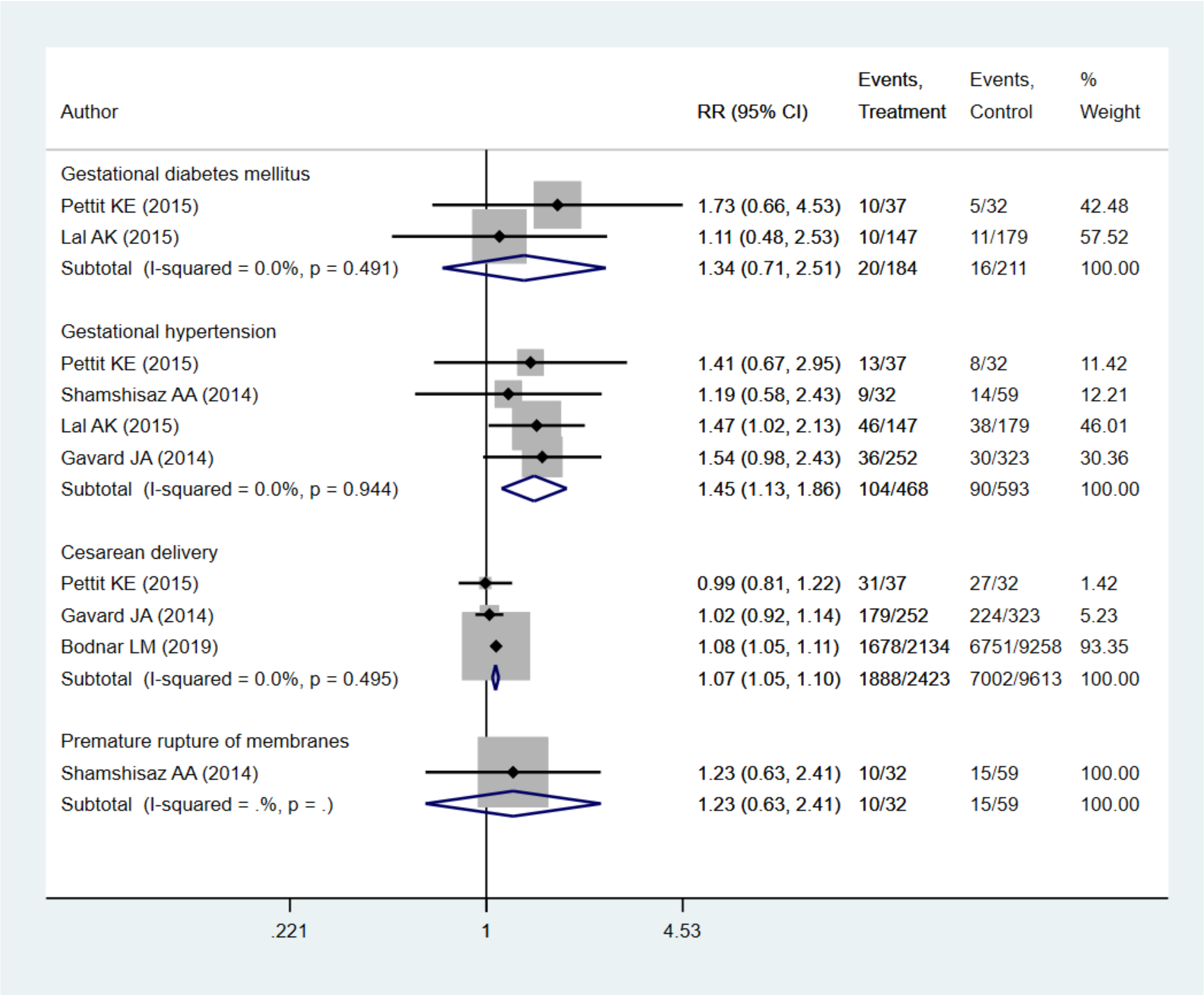

Supplementary Figure 16. Forest plot for neonatal outcomes in obese mothers (excessive weight gain vs. adequate weight gain)

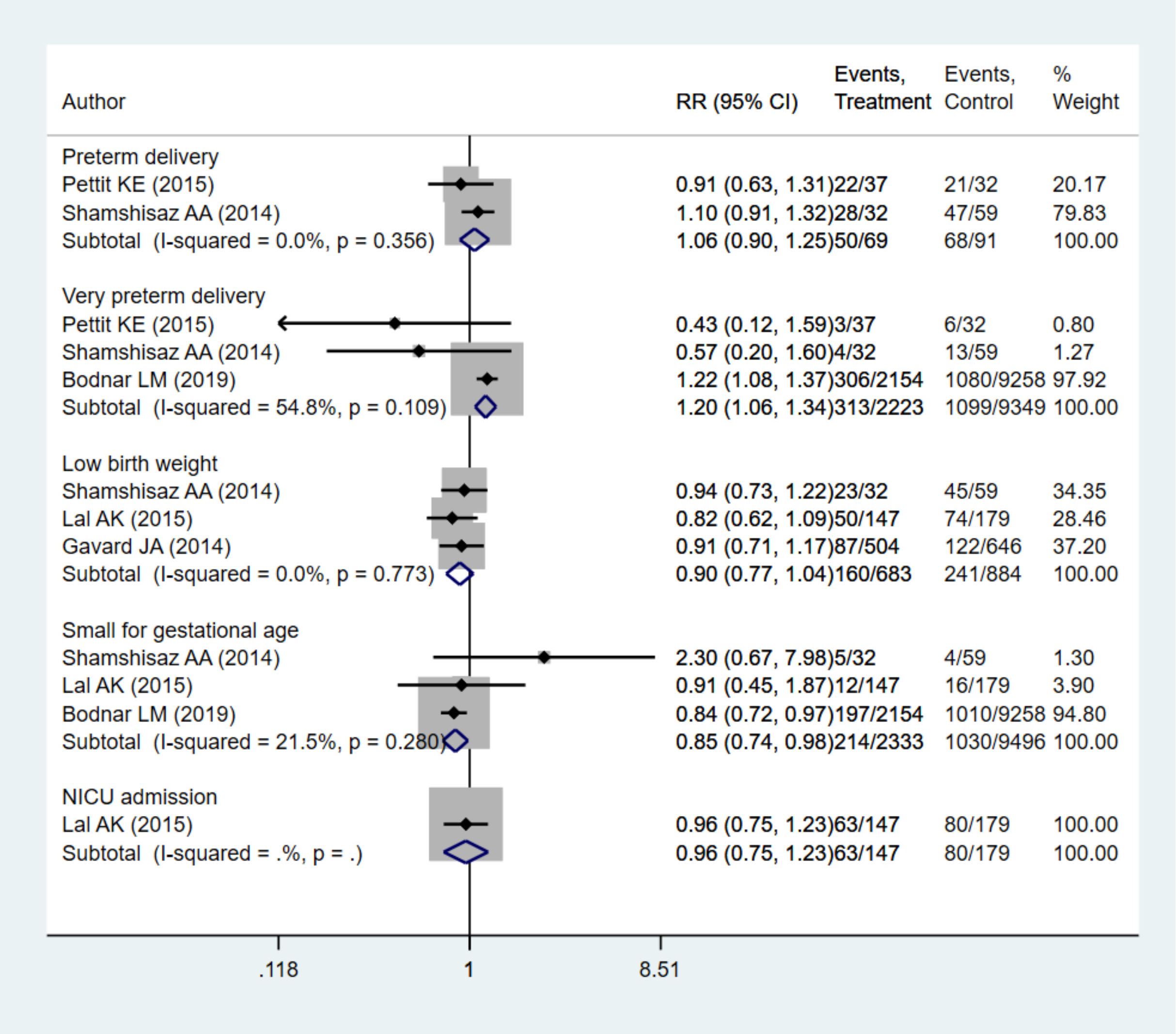

Supplement: Supplementary file 4 [file Data_Sheet_1.PDF]
